# Utilization of Quantitative In Vivo Pharmacology Approaches to Assess Combination Effects of Everolimus and Irinotecan in Mouse Xenograft Models of Colorectal Cancer

**DOI:** 10.1371/journal.pone.0058089

**Published:** 2013-03-08

**Authors:** Erica L. Bradshaw-Pierce, Todd M. Pitts, Gillian Kulikowski, Heather Selby, Andrea L. Merz, Daniel L. Gustafson, Natalie J. Serkova, S. Gail Eckhardt, Colin D. Weekes

**Affiliations:** 1 Division of Medical Oncology, University of Colorado Anschutz Medical Campus, Aurora, Colorado, United States of America; 2 Department of Anesthesiology, University of Colorado Anschutz Medical Campus, Aurora, Colorado, United States of America; 3 University of Colorado Cancer Center, University of Colorado Anschutz Medical Campus, Aurora, Colorado, United States of America; 4 Department of Clinical Sciences and Animal Cancer Center, Colorado State University, Fort Collins, Colorado, United States of America; Bauer Research Foundation, United States of America

## Abstract

**Purpose:**

The PI3K/AKT/mTOR pathway is frequently dysregulated in cancers and inhibition of mTOR has demonstrated the ability to modulate pro-survival pathways. As such, we sought to determine the ability of the mTOR inhibitor everolimus to potentiate the antitumor effects of irinotecan in colorectal cancer (CRC).

**Experimental Design:**

The combinatorial effects of everolimus and irinotecan were evaluated *in vitro* and *in vivo* in CRC cell lines harboring commonly found mutations in *PIK3CA*, *KRAS* and/or *BRAF*. Pharmacokinetically-directed dosing protocols of everolimus and irinotecan were established and used to assess the in vivo antitumor effects of the agents. At the end of treatment, 3–6 tumors per treatment arm were harvested for biomarker analysis by NMR metabolomics.

**Results:**

Everolimus and irinotecan/SN38 demonstrated synergistic anti-proliferative effects in multiple CRC cell lines *in vitro*. Combination effects of everolimus and irinotecan were determined in CRC xenograft models using clinically-relevant dosing protocols. Everolimus demonstrated significant tumor growth inhibition alone and when combined with irinotecan in HT29 and HCT116 tumor xenografts. Metabolomic analysis showed that HT29 tumors were more metabolically responsive than HCT116 tumors. Everolimus caused a decrease in glycolysis in both tumor types whilst irinotecan treatment resulted in a profound accumulation of lipids in HT29 tumors indicating a cytotoxic effect.

**Conclusions:**

Quantitative analysis of tumor growth and metabolomic data showed that the combination of everolimus and irinotecan was more beneficial in the *BRAF/PIK3CA* mutant HT29 tumor xenografts, which had an additive effect, than the *KRAS/PIK3CA* mutant HCT116 tumor xenografts, which had a less than additive effect.

## Introduction

There is currently significant focus on the development of novel agents designed to perturb signal transduction pathways important in cancer progression. The clinical use of these molecularly targeted agents frequently involves combinations with other therapeutic modalities and several clinical studies have demonstrated the benefit of adding signal transduction modulators (STMs) to chemotherapy or radiation therapy [1]–[5]. The successful development of drug therapies and treatment strategies requires the thoughtful use of preclinical models and careful interpretation of data.

The use of quantitative approaches, including the use of pharmacokinetic data and quantitative measures of response, is critical for elucidating mechanisms of action and improving translational pharmacology research [6]. A common shortcoming of *in vivo* pharmacology studies is the use of doses and/or schedules that are not clinically feasible which can lead to misleading results of efficacy and/or development of biomarkers that often fail to translate to the clinical setting. Here we present a study where we quantitatively determined the benefit of adding a small molecule STM, everolimus (Novartis, East Hanover, NJ), to standard chemotherapy, irinotecan (Pfizer Inc, New York, NY), using doses and schedules in our preclinical models predicted to yield drug exposures approximating those observed in patients.

Everolimus (40-*O*-(2-hydroxyethyl)-rapamycin, RAD001/Afinitor®) is an orally bioavailable inhibitor of the mammalian target of rapamycin (mTOR) which is currently approved for the treatment of advanced renal cell carcinoma and progressive neuroendocrine tumors of pancreatic origin (PNET) [7], [8]. mTOR, a serine/threonine kinase, is a central regulator of pathways that signal growth, proliferation, survival, metabolism and angiogenesis [7], [9]. mTOR activity is mediated by growth factor signaling, nutrient and energy states as well as hypoxic stress. Furthermore, mTOR plays a key role in the phosphatidylinositol 3-kinase (PI3K)/AKT pathway which is frequently dysregulated and implicated in the growth and progression in several cancers, making it an attractive therapeutic target [10], [11]. Recent studies suggest that PIK3CA mutations or AKT activity confer sensitivity to mTOR therapy [12]–[15]. *PIK3CA* is the gene that encodes the PI3K p110 catalytic subunit and mutations (exon 9 and exon 20) can be found in 10–30% of CRC [11], [16], [17].

CRC is the third most common cancer type, accounts for nearly 10% of all cancer-related deaths in the U.S [18]. While early stage CRC has a favorable 5-year survival rate, late stage disease with distant metastases has a 5-year survival rate of only 10%, indicating the need for improved treatment regimens for metastatic CRC (mCRC). Irinotecan (Camptosar®) is a standard of care chemotherapeutic agent used for the treatment of mCRC. We hypothesized that everolimus would enhance irinotecan therapy due to the modulation of effectors on pro-survival pathways and aimed to evaluate the combination in mouse xenograft models of CRC harboring the difficult to treat concurrent *PIK3CA* and *KRAS* or *BRAF* mutations. Additionally, because of the known metabolic effects of mTOR pathway inhibition, we quantitatively assessed the metabolic profiles of the tumors treated with clinically relevant doses of everolimus and irinotecan by nuclear magnetic resonance (NMR) spectroscopy.

## Materials and Methods

### Chemicals and Reagents

Irinotecan for *in vivo* studies was obtained from the University of Colorado Hospital Pharmacy (Aurora, CO) and SN38 for *in vitro* studies was purchased from LKT labs (St. Paul, MN). Everolimus was provided as a suspension by Novartis. SN38 stock solutions for *in vitro* experiments were made in DMSO (Fisher Scientific, Pittsburgh, PA). All other materials used were purchased from either Fisher Scientific or Sigma (St Louis, MO) unless otherwise specified.

### Cell Culture

Colon tumor cell lines, HCT8, HT29, LS180 and HCT116, were purchased from the American Type Culture Collection, (Manassas, VA), and maintained on tissue culture plates (BD Falcon, San Jose, CA) in RPMI (Gibco) supplemented with 10% fetal bovine serum (Gibco) and penicillin (100 units/mL)-streptomycin (100 µg/mL; Life Technologies). Cells were routinely screened for mycoplsma using MycoAlert (Lonza). All cells were maintained at 37°C in a humidified incubator with 5% CO_2_. All *in vitro* drug treatments were conducted with the use of complete growth medium.

### Cytotoxicity and Combination Effects

Cytoxic effects were determined using the sulforhodamine B (SRB) assay. Briefly, 5000 viable cells were plated into 96-well plates and incubated overnight prior to exposure with different concentrations of drugs. Cells were exposed to increasing concentrations of everolimus (0–200 nM), SN38 (0–8 nM), and combinations of the two. Following a 72 hour incubation, media was removed and cells were fixed with cold 10% trichloroacetic acid for 30 minutes at 4° C. Cells were washed with water and stained with 0.4% SRB for 30 minutes at room temperature. Cells were washed again with 1% acetic acid and stain was solubilized with 10 mM tris at room temperature and read at an OD of 565 nm. The results of the combined treatment were analyzed according to the isobolographic method of Chou and Talalay, using the Calcusyn software program (Biosoft, Cambride, UK). The resulting Combination Index (CI) was used as a quantitative measure of the degree of interaction between different drugs. A CI value equal to 1 denotes additivity; CI greater than 1, antagonism; CI less than 1, synergism.

### Immunoblotting

Cells were seeded into 6-well plates 24 hours prior to treatment with each drug alone or in combination for 24 hours. Cells were scraped into RIPA buffer containing protease inhibitors, EDTA, NaF, and sodium orthovanadate. Total protein was determined using the Pierce 660 nm Protein Assay (Pierce, Rockford, IL). Thirty micrograms of total protein was loaded onto a 4–12% gradient gel, electrophoresed and transferred to nitrocellulose using the iBlot system (Invitrogen, Carlsbad, CA). Membranes were blocked for one hour at room temperature with Licor Blocking Buffer (Licor, Lincoln, NE) prior to overnight incubation at 4°C with one of the following antibodies: pS6RP, tS6RP, pAKT, tAKT, pERK, tERK, p21, PARP, actin and α-tubulin (Cell Signaling, Beverly, MA). All antibodies were used at a 1∶1000 dilution except for pERK, which was used at 1∶2000. Following primary antibody incubation, blots were washed in TBS-Tween(0.1%), then incubated with the appropriate secondary antibody at 1∶15,000 (Licor, Lincoln, NE) for one hour at room temperature. After three additional washes, blots were developed using the Licor Odyssey (Licor, Lincoln, NE).

### Animals

Female athymic nude mice, 5 to 10 weeks old, were purchased from the National Cancer Institute. Animals were housed 3–5 per cage in polycarbonate cages and kept on a 12 h light/dark cycle. Food and water were given *ad libitum*. All studies were approved by the Institutional Animal Care and Use Committee (IACUC) and conducted in accordance with the NIH Guidelines for the Care and Use of Laboratory Animals, and animals were housed in a facility accredited by the American Association for Accreditation of Laboratory Animal Care.

For tumor bearing studies, cells were harvested and resuspended in a 1∶1 mixture of serum-free RPMI and Matrigel (BD Bioscience). One million cells were injected subcutaneously into each rear flank of mice. Tumor volumes, measured by digital calipers, were calculated by *V* (mm^3^) = length x (width)^2^ x 0.5236.

### Pharmacokinetic Study

A pharmacokinetic study of everolimus was conducted in mice bearing HT29 tumors (average volume ∼300 mm^3^). Mice were treated with either 2.5 or 10 mg/kg everolimus daily for 7 days by oral gavage. Three mice per dose level were sacrificed by cardiac stick exsanguination at 30 minutes, 2, 4, 6 and 24 hours following drug administration on day 7. Plasma and tumor tissue were snap-frozen in liquid N_2_ and stored at −70°C until analyzed.

Everolimus was measured in plasma and tumors by LC/MS/MS assay. Analytical standards, quality control (QC) and unknowns were all prepared by adding 200 µl of unknown or spiked blank plasma or tumor homogenate (100 mg/ml in water) samples to a 1.5 ml microcentrifuge tube. Samples were extracted by the addition of 1 ml methyl tert-butyl ether (MTBE) followed by 10 min vortex mixing. Samples were centrifuged at 13,000 RPM for 10 min and the supernatant collected and 900 µl transferred to a clean glass test tube. Samples were evaporated to dryness using a rotary evaporator followed by re-suspension in 100 µl of 50% acetonitrile/50% 10 mM ammonium acetate and transferred to auto-sampler vials for analysis. Mass spectra for extracted samples was obtained on a MDS Sciex 3200 Q-TRAP triple quadrupole mass spectrometer (Applied Biosystems, Inc., Foster City, CA) with a turbo ionspray source interfaced to an Agilent 1200 Series Binary Pump SL HPLC system (Santa Clara, CA).The lower and upper limits of quantitation for the assay were 1 and 5000 nM, respectively. Accuracy and precision (% RSD) based on analysis of standards and QC samples for this assay were 90.7% and 5.5% for plasma and 93.0% and 4.2% for tumor. Additional details may be found in Supplement S1 (section SA.1.).

### Therapeutic Study

When HT29 and HCT116 tumors reached an average volume of ∼325 mm^3^ animals were randomized into four treatment groups (n = 9–10 mice per group): (i) vehicles, (ii) 5 mg/kg everolimus (RAD) by oral gavage (PO) daily, (iii) 10 mg/kg irinotecan (IRI) once weekly by intravenous (iv) tail vein injection, and (iv) a combination of RAD and IRI. Everolimus, diluted in sterile water, and irinotecan, diluted in sterile 0.9% saline, were administered at 4 mL/kg. Animals were treated for 28 days. Tumor volumes and body weights were measured 2–3 times weekly. On day 28 of treatment 3–6 animals per group were sacrificed, approximately 24 hours following everolimus treatment, and tumors harvested for metabolomic analysis.

### NMR-Based Metabolomics

For metabolomic analysis, animals were fasted for 4 hours then received 250 mg/kg of [1-^13^C] glucose (Cambridge Isotopes, Cambridge, MA) by IV injection 60 minutes prior to tumor harvesting. Snap-frozen tumor specimens underwent two-phase acid extraction procedure (using 8% perchloric acid) previously established and extensively published [19]–[22]. Further details may also be found in Supplement S1 (section SA.2.).

### Data Analysis

Plasma and tumor pharmacokinetic parameters were calculated by noncompartmental analysis with WinNonlin. Everolimus plasma data was fit to a two-compartment model with first-order absorption and simulations of plasma concentrations at a 5 mg/kg dose of everolimus were performed by SAAM II version 2.1 (The Epsilon Group, Charlottesville, VA/University of Washington).

One-way ANOVA analyses with a Tukey post-test was used to determine statistical significance between multiple groups. Analyses were performed with Prism version 4.02. *P* values<0.05 were considered statistically significant. All quantitative metabolomic data sets were included in custom-built metabolic fluxes analyzers and data interfaces and presented as metabolic heat maps [23].

Data from the in vivo study was modeled to assess the therapeutic benefit of adding everolimus to irinotecan. The model used was similar to a previously published model for assessing combination therapy [24]. Some modifications were made and details of the model can be found in Supplement S1 (section SA.3.) and Supplement S2. All modeling was performed on individual animal tumor volume data and modeled with SAAM II version 2.1.

## Results

### In Vitro Activity

**Figure 1:**
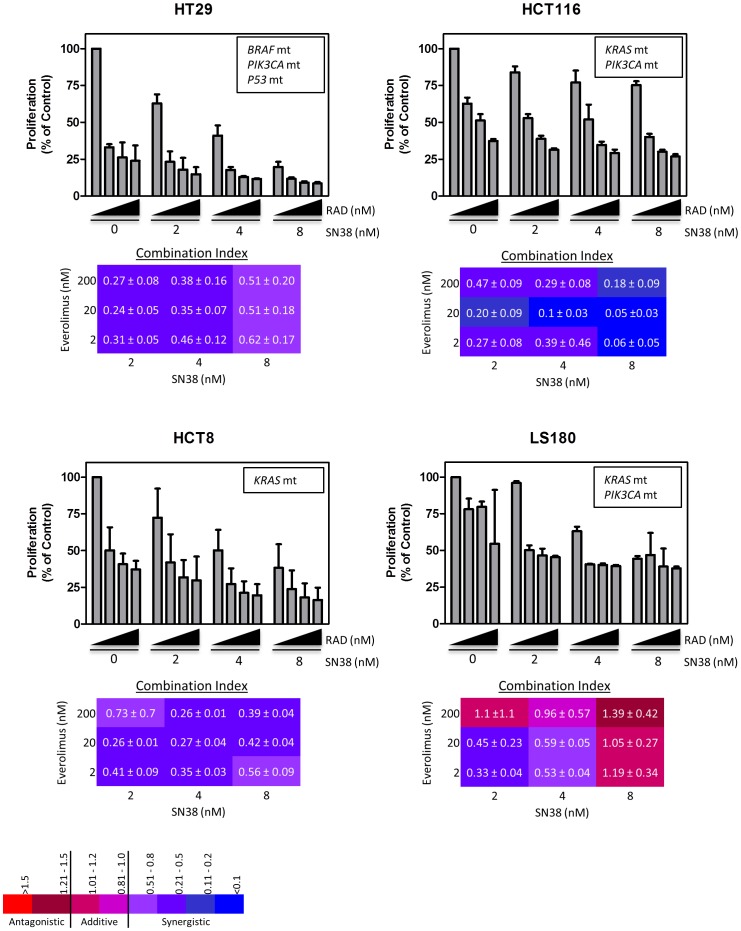
*In vitro* anti-proliferative effects of everolimus (RAD) and SN38 combinations in colorectal cancer cell lines. The combinatorial anti-proliferative effects were evaluated in HT29, HCT116, HCT8 and LS180 cells to assess potential additive or synergistic interactions. Growth inhibition was measured by the sulforhodmine B assay (SRB) following a 72 hour incubation with SN38 (0, 2, 4 or 8 nM) and RAD (0, 2, 20 or 200 nM - indicated by the triangels under the graphs). Data on the graphs represents the mean ± standard deviation of at least 3 separate experiments. The combination index (CI) values were calculated for for all combinations and the values ± the standard deviation are presented in the colored tables below each graph.. The %CV for replicates ranged from 5–40% and averaged ∼20% therefore, we determined that CI values>1.2 are considered antagonistic, 1.2> CI>0.8 are additive, and CI<0.8 are synergistic. Note that SN38 is used in *in vitro* assays instead of irinotecan since it is the active metabolite.

**Figure 2:**
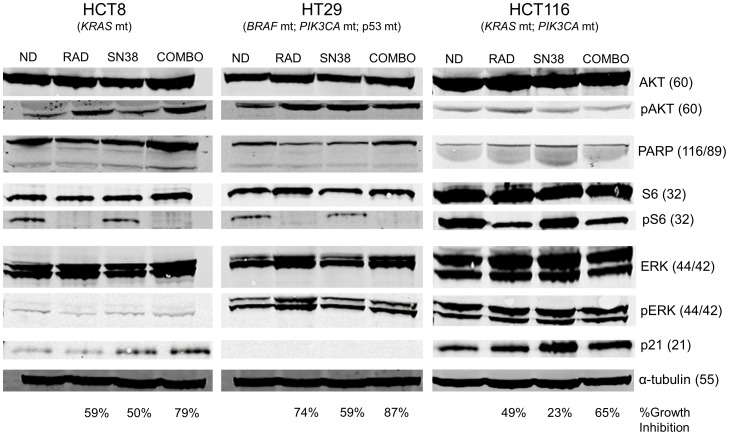
Western blot analysis of everolimus and SN38 treatment effects in HCT8, HT29 and HCT116 cells. Akt, p-Akt, PARP, cleaved PARP, total ribosomal S6 kinase (S6), ribosomal phospho-S6 kinase (pS6), ERK, p-ERK, p21 and α-tubulin were measured in all lines following 24 hours of treatment with RAD (20 nM), SN38 (4 nM) or the combination of the two. Molecular weights are presented next to protein name in parenthesis. Note that SN38 is used in *in vitro* assays instead of irinotecan since it is the active metabolite.

The synergistic effects of everolimus (RAD) and SN38 were assessed in CRC cell lines, HT29 (*BRAF* mt; *PIK3CA* mt; p53 mt), HCT116 (*KRAS* mt, *PIK3CA* mt), HCT8 (*KRAS* mt) and LS180 (*KRAS* mt; *PIK3CA* mt). For all *in vitro* experiments SN38 was used in place of irinotecan since SN38 is the active metabolite of irinotecan. HT29 cells were the most sensitive to SN38 and everolimus and the addition of everolimus to SN38 generally provided a strongly synergistic effect in HT29, HCT8 and HCT116 cells with CI values ranging from<0.1 to 0.7 (Figure 1). The addition of everolimus to SN38 treatment in LS180 cells was less clear as CI values ranged widely from 0.3–1.4. Western blot analyses show that at low concentrations, everolimus (20 nM) is able to inhibit ribosomal phospho-S6 kinase (pS6RP) in all cell lines and SN38 (4 nM) causes an increase in p21 in HCT116 and HCT8 cells (Figure 2). No p21 was measurable in HT29 cells at doses of everolimus and irinotecan used. A small amount of cleaved PARP (MW 89) was observed in SN38 treated HCT8 cells, but not in HT29 or HCT116.

### Pharmacokinetic-Directed Dosing of Everolimus and Irinotecan

For *in vivo* studies, we established doses of everolimus and irinotecan to administer to mice that would yield exposures at or below clinically achievable levels. Human pharmacokinetic information on everolimus [25], [26] and irinotecan/SN38 [27]–[30] was gathered from the literature and is presented in Tables 1 and 2 respectively.

**Table 1:** Pharmacokinetic parameters for everolimus^a^ in humans and mice.

	Dose	Species	t_½_ (hr)	AUC_0→t_ (ng·hr/mL)	C_max_ (ng/mL)	C_min_ (ng/mL)	Ref.
*Everolimus* a							
	10 mg	Human	–	514±231	61±17^b^	13.3^b^	[25]
	10 mg	Human	–	711±113	66±1.4^b^	∼20^bd^	[26]
	5 mg	Human	–	238±77	32±9^b^	^†^5.4	[25]
	5 mg	Human	–	543±189	58±18^b^	∼12–14^bd^	[26]
	10 mg/kg	Mouse	5.0	11592	1684±277^b^	59±5^b^	
	2.5 mg/kg	Mouse	6.0	1921	350±230^b^	12±6^b^	
	5 mg/kg^e^	Mouse	5.7	3716	561^bc^	21^b^	

a All PK data obtained for everolimus is for daily oral administration.

b Steady-state parameter.

c Value of C_max_ for simulated data is taken at 0.5 h for comparison to data measured at this time.

d Data estimated from graph in references.

e Data for 5 mg/kg RAD001 is based on simulations.

**Table 2:** Pharmacokinetic parameters for Irinotecan/SN38 in humans and mice.

Dose	Species	Schedule	t_½_ (hr)	AUC_0→t_(ng·hr/mL)	C_max_(ng/mL)	Ref.
*Irinotecan/SN38 (PK data is for SN38)*						
125mg/m^2^	Human	1.5 hr INF	10.4±3.1	229±108	26.3±11.9	[27]
340mg/m^2^	Human	1.5 hr INF	21.0±4.3	474±245	56.0±28.2	[27]
350mg/m^2^	Human	1 hr INF	23±35	931±948	88±26	[28]
350mg/m^2^	Human	1 hr INF	15±23^a^	609±531^a^	57±20^a^	[28]
125mg/m^2^	Human	1.5 hr INF	28.5	228±149	27±12	[29]
180mg/m^2^	Human	1.5 hr INF		222–245^a^		[30]
5mg/kg	Mouse	IV Bolus		279^c^	647	[39]
10mg/kg	Mouse	IV Bolus		534^c^	443	[39]
10mg/kg	Mouse	IV Bolus		533–759^c^	435–600^b^	[37]
10mg/kg	Mouse	IV Bolus		533–1029^c^	443–673^b^	[36]
10mg/kg	Mouse	IV Bolus	2.2	410±60 (t = 6 h)		[36]
20mg/kg	Mouse	IV Bolus	3.0	710±240 (t = 6 h)		[36]
40mg/kg	Mouse	IV Bolus	3.4	1080±110 (t = 6 h)		[36]

a Week 4 PK with cetuximab.

b Data estimated from graph in references.

c AUC_0→∞_was reported.

To determine the appropriate dose of everolimus in mice, we conducted a pharmacokinetic study of everolimus in HT29 tumor bearing mice. Steady-state plasma and tumor concentration-time profiles are presented in Supplement S3 Figures SC.2. A & B and non-compartmental pharmacokinetic parameters presented in Table 1 and Table C.1 in Supplement S3. The data showed that a dose of 10 mg/kg in mice results in steady-state free plasma exposure (AUC_ss,free_ = 116 ng*hr/mL) which is in the range of that observed in humans at the clinically used doses of 5 to 10 mg/kg per day (AUC_ss,free_ = 60–178 ng*hr/mL). Plasma protein binding values of 99% and 75% were used to calculate free plasma concentrations in mice and humans respectively [31], [32]. However, for the in vivo efficacy studies, we opted to use a dose of 5 mg/kg (AUC_ss,free_ = 37 ng*h/mL) being cautious of achieving too much single agent activity, which could make it difficult to assess combination effects. Previous studies of everolimus in mice bearing HCT116 tumor xenografts demonstrated single agent activity (45–55% tumor growth inhibition) of everolimus at 10–12 mg/kg [33], [34].

*In vivo* SN38 data was evaluated to define the dose of irinotecan to be used since it is the active metabolite of irinotecan and carboxylesterase conversion of irinotecan to SN38 varies between mice and humans. Based on human and mouse pharmacokinetic data gathered from literature data [27]–[30], [35]–[40], we selected a dose of 10 mg/kg of irinotecan to be administered intravenously once weekly (Table 2). A 10 mg/kg iv dose was estimate to yield a free exposure of SN38 in the range of that observed in humans (mice: AUC_free_ = 7.5–25.8 ng*hr/mL; humans: AUC_free_ = 4.4–18.6 ng*hr/mL for doses ranging from 125–350 mg/m^2^). SN38 plasma protein binding values of 96.6–98.6% for mice and 98% for humans were used to calculate free fraction from total concentrations [37].

### Tumor Xenograft Response to Everolimus and Irinotecan Therapy

**Figure 3:**
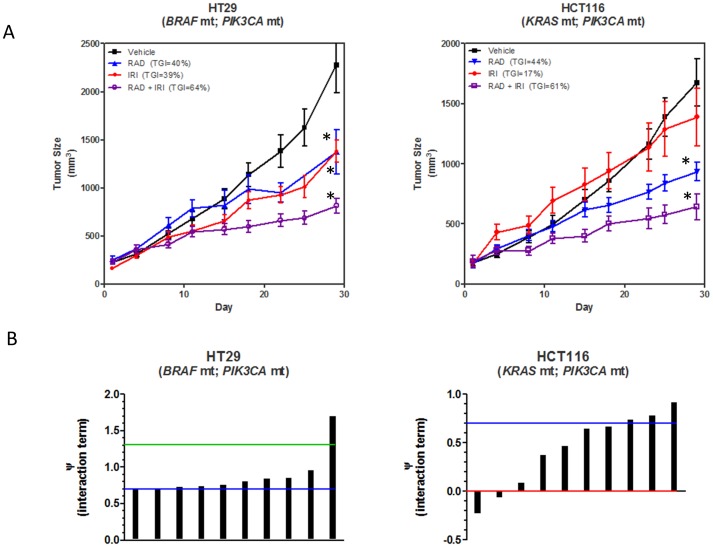
*In vivo* effects of everolimus (RAD), irinotecan (IRI) and combination of the two agents on CRC tumor xenografts. (A) HT29 and HCT116 tumor xenograft growth curves. Animals were treated for 28 days with vehicles, RAD, IRI, or the combination of RAD+IRI. Data represents the average ± SEM of 9–12 tumors per group. ^*^P<0.05 versus vehicle. (B) Pharmacokinetic-pharmacodynamic modeling was performed on to quantitatively assess the intensity of the RAD+IRI combination in HT29 and HCT116 tumor xenografts. This is a graphical representation of the interaction term (ψ) for the RAD+IRI combination. Each bar represents the ψ value for an individual tumor. ψ values>1.3 are synergistic, 1.3> ψ>0.7 are additive, 0.7> ψ>0 are less than additive, and ψ<0 are antagonistic.

The efficacy of everolimus, irinotecan and combination of the two was determined in HT29 and HCT116 tumor xenografts. Figure 3A shows the tumor growth profiles of all treatment groups for both tumor types. Everolimus caused similar and statistically significant (p<0.05 vs. control) growth inhibition in both HT29 (average tumor growth inhibition, TGI = 40%) and HCT116 (average TGI = 44%) tumors whereas irinotecan caused significant growth inhibition in only HT29 tumors (average TGI = 39%; p<0.05). The addition of everolimus to irinotecan significantly reduced the volume of HT29 (average TGI = 64%) and HCT116 (average TGI = 61%) tumor xenografts (P<0.05 versus control).

Modeling of the tumor growth profiles and effect of treatment revealed that the combination of everolimus and irinotecan was on average additive (average ψ = 0.9) in HT29 tumors (Figure 3B). Each tumor was modeled individually and of the 10 HT29 tumors, 1 showed a synergistic response, 8 showed an additive response and 1 was less than additive, but not antagonistic. ψ represents the term used in the mathematical model to describe the combinatorial effect, where ψ>1.3 is synergistic; 1.3> ψ>0.7 represents an additive interaction, 0.7> ψ>0 is a less than additive effect and ψ<0 is antagonistic. Details on the modeling and individual tumor data and fits can be found in the Supplement S1, section SA.3., and Supplement S2. The growth profiles of the HCT116 xenografts were much more variable than the HT29 tumors. Therefore, although the average tumor growth inhibition of the combination treatment appears similar in the two tumor types (TGI = 64% vs 61%), in HCT116 tumors the benefit was on average less than additive (average ψ = 0.5). Again, each tumor growth profile was modeled individually and 3 tumors showed an additive effect, 5 were less than additive and 2 were antagonistic.

**Figure 4:**
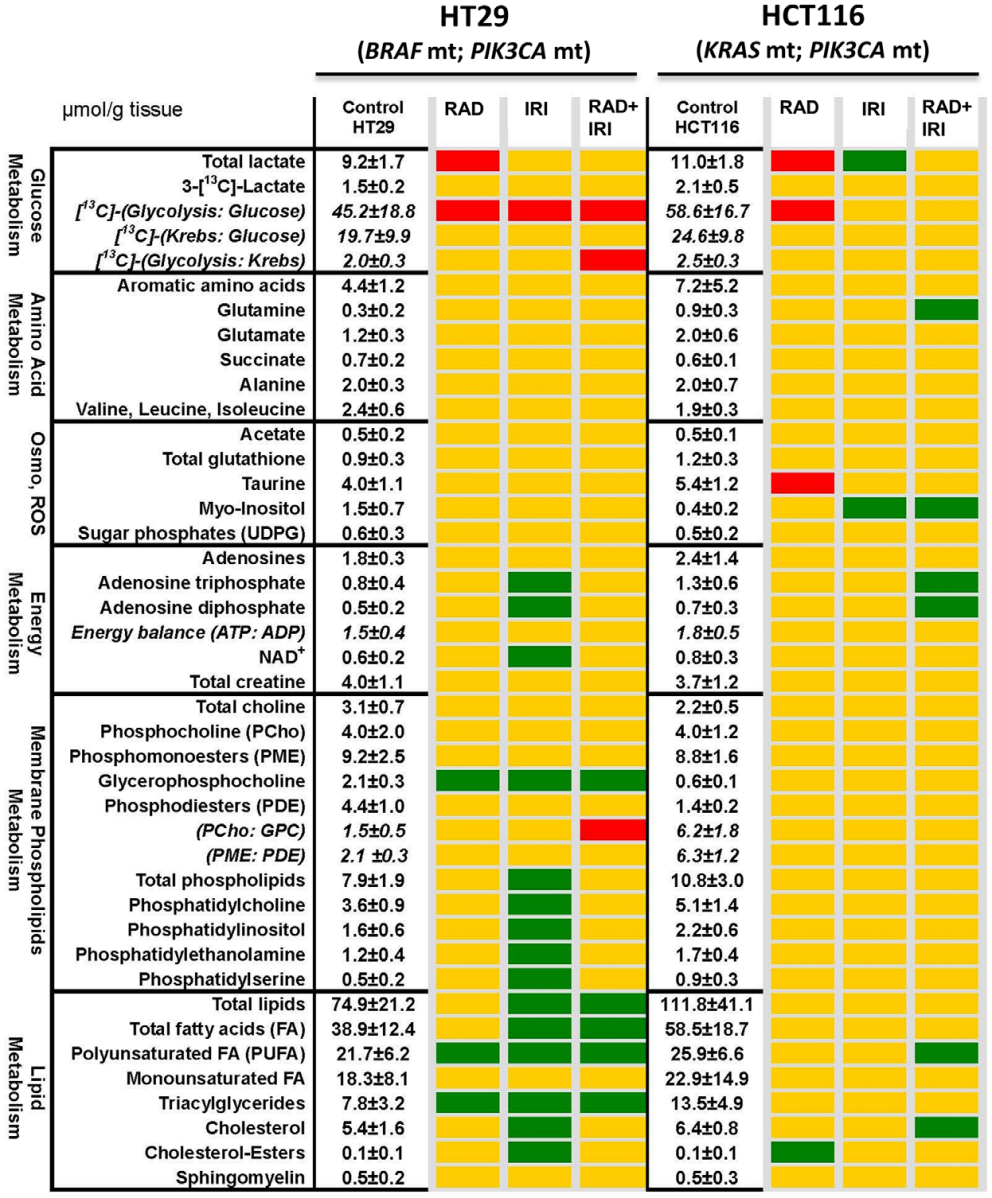
Metabolic heat-maps based on quantitative NMR spectroscopic data sets in HT29 and HCT116 xenografts at the end of the study. ^1^H-, ^13^C-, and^ 31^P-NMR data are represented as mean ± SEM of 3–6 measurements. The metabolites, their ratios and metabolic fluxes were grouped based on their biochemical relevance. For the control group, all intracellular metabolite levels are given as µmol per gram cell wet weight and metabolite ratios are unitless. Metabolic pathways which were undisturbed by treatment are presented as yellow maps. A decrease in metabolic end-point is indicated by red, while an increase by green spots. Statistical significance for metabolite changes are based on multivariate analysis of metabolic fluxes with p<0.02. The interactive metabolic profile array database was custom-based [23].

The effects of everolimus, irinotecan and the combination of the two on tumor metabolism were measured in a subset of animals at the end of the study. Metabolic profiles were determined by ^1^H-, ^13^C- and ^31^P- NMR. Figure 4 shows metabolic heat-maps to depict metabolic changes among treatment groups relative to the control group in HT29 and HCT116 xenografts. The effects of everolimus on glucose metabolism, decrease in lactate and glycolysis, were similar in HT29 and HCT116 tumors, consistent with the tumor growth response observed in the two tumor types. HT29 tumors showed a more profound response to irinotecan treatment, which is mostly related to accumulation of lipids, especially poly-unsaturated and phospholipids, which can be related to increased cellular degradation and necrosis. Again, this is consistent with the tumor growth profiles of HT29 and HCT116 tumors which showed that HT29 tumors responded to irinotecan treatment while HCT116 tumors did not. The combination of everolimus with irinotecan was associated with increased lipid accumulation, membrane degradation (increased GPC and decreased PC/GPC) and to some degree decreased glycolysis in HT29 tumors. The same response was not observed in HCT116 tumors, despite the average tumor growth inhibition of the combination treatment yielding roughly the same result. Overall, HT29 tumors were more metabolically responsive to all treatment than HCT116 tumors.

## Discussion

The development of new therapeutics and therapeutic strategies requires the use of preclinical models. Frequently, the impressive preclinical activity of promising new agents and combinations of agents does not translate to viable clinical treatments. Rational study design with quantitative endpoints is critical for the effective translation of combination strategies and biomarkers of efficacy [6], [41]. The overall goal of the study described herein was to quantitatively establish the combinatorial effect of everolimus and irinotecan therapy in murine models of CRC, harboring difficult to treat genotypes, utilizing pharmacokinetic-guided dosing regimens. Based on human and mouse pharmacokinetic information, we established doses of everolimus and irinotecan to be used in preclinical *in vivo* studies that yield exposures in the range of those observed in humans. It is important to note that we corrected total concentrations for the free or unbound fractions when making comparisons of pharmacokinetic data between mice and humans. This is critical since the plasma protein binding often varies between species and in theory only the unbound fraction of drug is available for interaction with the target of interest.

We tested the everolimus and irinotecan combination *in vivo* in two cell line xenograft models of CRC, HT29 and HCT116. The combination was well tolerated as significant bodyweight changes were not observed (Supplement S3 Figure SC.2.). Evaluation of the end of study tumor data from each tumor type shows that treatment with everolimus and irinotecan lead to statistically significant tumor growth inhibition and the degree of response appears similar in the two types. However, here we show that by evaluating each tumor’s growth profile, the volume as a function of time, we are able to elucidate differences in the growth and response between the two tumor types. Through mathematical modeling of the control, single agent and combination arms of the study we were able to quantitatively assess the interaction of the two compounds and found that in the HT29 tumors, *BRAF* and *PIK3CA* mutant, the combination was additive and in HCT116 tumors, *KRAS* and *PIK3CA* mutant, the combination was quite variable but less than additive on average. In addition to evaluating tumor growth we quantitatively assessed the effects of the treatments on tumor metabolism using NMR-based metabolomics.

Metabolically, HT29 xenografts were more responsive than HCT116 xenografts. HT29 tumors, but not HCT116 tumors, in the irinotecan group displayed a typical metabolic signature for cytotoxic therapy, accumulation of lipids and phospholipids, an indication of cytotoxicity/necrosis [42]. This result is consistent with the tumor growth profiles, which shows HT29 tumors responding to irinotecan treatment (TGI = 39%, p<0.05) but not HCT116 tumors (TGI = 17%). These results also agree with the *in vitro* data, which show HT29 cells (IC50 = 3 nM) being more sensitive than HCT116 cells (IC50>300 nM) to SN38 treatment. According to our simulations (Supplement S2), the free concentrations of SN38 in the plasma ranged from 1300 to 7.25e−4 nM in a 24 hour period with an average concentration of 4–18 nM, which is well below the estimated IC50 for HCT116 cells. Despite the similarity in tumor growth inhibition profiles of everolimus treated HT29 tumors and HCT116 tumors (TGI = 41% vs 44%, respectively), HT29 tumors appeared slightly more metabolically responsive than HCT116 tumors. Some decrease of glycolysis was measured in both HT29 and HCT116 tumors, but an increase in GPC and some lipid accumulation was also observed in HT29 tumors. In vitro, HT29 cells were more responsive to everolimus treatment than HCT116 cells, as the IC50 for proliferation was lower (1.2 nM vs. 25 nM) and greater p-S6RP inhibition was observed at 20 nM. *In vivo,* similar responses in inhibition of p-S6RP and cyclin D1 were observed in HT29 and HCT116 tumors (Supplement S3 Figure SC3 and SC4); however, measures of p-S6RP may not be a useful measure to assess efficacy. A previously published study showed that everolimus treatment in both sensitive and resistant lines can result in total dephosphorylation of S6K1 and S6 [33].

We speculate that some of the efficacy of everolimus observed in HCT116 tumors may be related to antiangiogenic effects of everolimus. Antiangiogenic effects of everolimus have been previously reported [33], [43] and antiangiogenic effects would be much harder to detect by whole tumor metabolomics, as performed here, since the fraction of endothelial cells compared to total tumor tissue is quite small. Further evidence to support this may be derived from everolimus pharmacokinetic data. The free plasma concentrations produced at the 5 mg/kg dose in our animals ranged from 0.2 to 6 nM (C_min_ to C_max_) with an average free concentration (C_ss,avg_) of about 1.5 nM, which would be sufficient to inhibit HT29 growth, but not HCT116 and, previous data report potent anti-proliferative activity of everolimus against VEGF and bFGF stimulated endothelial cells (HUVECs) with IC50 values from 0.1–0.8 nM [33]. In the combination group of the HT29 xenografts we did observe some additive metabolic effects, everolimus-based glycolytic inhibition and irinotecan-based lipid accumulation at the end of the study. Although some trends were apparent, there were few statistically significant alterations in the metabolomics of treated HCT116 tumors.

Overall, HT29 tumors, *in vitro* and *in vivo* were more responsive to everolimus, irinotecan/SN38 and combination therapy. In part, the reduced response of HCT116 cells to everolimus treatment may be attributed to intrinsic metabolic dysregulation. HCT116 cells have higher basal glycolysis compared to HT29 cells *in vitro* (Supplement S3 Figure SC.5.) and *in vivo* (significantly higher PCho:GPC and PME:PDE ratios in HCT116 controls than HT29 controls, which are considered as metabolic proliferative indexes) despite having similar doubling times of 24–28 hours [44], [45]. Additionally, HCT116 cells harbor the *KRAS* mutation, which has been demonstrated to confer resistance to everolimus in tumors that also harbor the *PIK3CA* mutation [15]. It has also been proposed that, given its involvement in the MAPK pathway, *BRAF* mutations will also confer resistance to mTOR inhibitors [46]. However, the more durable response we observed in the HT29 tumors may suggest that the difference in effect between *BRAF* and *KRAS* mutations in mTOR therapy requires more thorough investigation.

*KRAS* and *BRAF* mutations are associated with poor prognosis in CRC [16], [47] and treatment of tumors harboring these mutations following primary treatment failure remains an unmet medical need. Everolimus has demonstrated clinical activity as a single agent [48], [49] and has shown in preclinical models to potentiate the effects of several different chemotherapeutic compounds [50]. Here we quantified the benefit of adding the everolimus to irinotecan in *KRAS* and *BRAF* mutant CRC tumor models. We believe that through the use of rigorous quantitative approaches we are able to derive more meaningful and potentially more translational information. Our study here suggests that everolimus and irinotecan combinations may be a viable treatment option for patients with mCRC who have failed prior therapy. Our data, together with previously published data [33], [43], may also suggest that everolimus exerts an antiangiogenic effect, which could mean that tumor *KRAS* genotype may not preclude a patient from benefiting from everolimus therapy. However, we feel this is an area that requires further study.

While we were able to demonstrate significant effects on tumor growth inhibition with clinically-relevant doses in these models, we feel the results must be interpreted with caution. The tumors did continue to grow while on treatment and therefore we might expect our best result clinically to be a delay in time to progression or an increase in progression free survival.

## Supporting Information

Supplement S1 **Supporting methods.**
(PDF)

Supplement S2 **Pharmacokinetic and Synergy Modeling.**
(PDF)

Supplement S3 **Supporting figures & tables.**
(PDF)

## References

[1] Moore MJ, Goldstein D, Hamm J, Figer A, Hecht JR, et al (2007) Erlotinib plus gemcitabine compared with gemcitabine alone in patients with advanced pancreatic cancer: a phase III trial of the National Cancer Institute of Canada Clinical Trials Group. J Clin Oncol 25: 1960–1966. 17452677 10.1200/JCO.2006.07.9525

[2] Bonner JA, Harari PM, Giralt J, Cohen RB, Jones CU, et al (2010) Radiotherapy plus cetuximab for locoregionally advanced head and neck cancer: 5-year survival data from a phase 3 randomised trial, and relation between cetuximab-induced rash and survival. Lancet Oncol 11: 21–28. 19897418 10.1016/S1470-2045(09)70311-0

[3] Suntharalingam M, Kwok Y, Goloubeva O, Parekh A, Taylor R, et al (2012) Phase II study evaluating the addition of cetuximab to the concurrent delivery of weekly carboplatin, paclitaxel, and daily radiotherapy for patients with locally advanced squamous cell carcinomas of the head and neck. Int J Radiat Oncol Biol Phys 82: 1845–1850. 21601372 10.1016/j.ijrobp.2011.02.062 PMC4447113

[4] Ocvirk J, Brodowicz T, Wrba F, Ciuleanu TE, Kurteva G, et al (2010) Cetuximab plus FOLFOX6 or FOLFIRI in metastatic colorectal cancer: CECOG trial. World J Gastroenterol 16: 3133–3143. 20593498 10.3748/wjg.v16.i25.3133 PMC2896750

[5] Giantonio BJ, Catalano PJ, Meropol NJ, O’Dwyer PJ, Mitchell EP, et al (2007) Bevacizumab in combination with oxaliplatin, fluorouracil, and leucovorin (FOLFOX4) for previously treated metastatic colorectal cancer: results from the Eastern Cooperative Oncology Group Study E3200. J Clin Oncol 25: 1539–1544. 17442997 10.1200/JCO.2006.09.6305

[6] Gabrielsson J, Green AR (2009) Quantitative pharmacology or pharmacokinetic pharmacodynamic integration should be a vital component in integrative pharmacology. J Pharmacol Exp Ther 331: 767–774. 19779129 10.1124/jpet.109.157172

[7] Houghton PJ (2010) Everolimus. Clin Cancer Res 16: 1368–1372. 20179227 10.1158/1078-0432.CCR-09-1314 PMC3003868

[8] Phan AT (2012) Metastatic pancreatic neuroendocrine tumors (pNET): Placing current findings into perspective. Cancer Treat Rev. 10.1016/j.ctrv.2012.02.010 22459199

[9] Dancey J (2010) mTOR signaling and drug development in cancer. Nat Rev Clin Oncol 7: 209–219. 20234352 10.1038/nrclinonc.2010.21

[10] Courtney KD, Corcoran RB, Engelman JA (2010) The PI3K pathway as drug target in human cancer. J Clin Oncol 28: 1075–1083. 20085938 10.1200/JCO.2009.25.3641 PMC2834432

[11] Samuels Y, Wang Z, Bardelli A, Silliman N, Ptak J, et al (2004) High frequency of mutations of the PIK3CA gene in human cancers. Science 304: 554. 15016963 10.1126/science.1096502

[12] Meric-Bernstam F, Akcakanat A, Chen H, Do KA, Sangai T, et al (2012) PIK3CA/PTEN mutations and Akt activation as markers of sensitivity to allosteric mTOR inhibitors. Clin Cancer Res 18: 1777–1789. 22422409 10.1158/1078-0432.CCR-11-2123 PMC3307149

[13] Weigelt B, Warne PH, Downward J (2011) PIK3CA mutation, but not PTEN loss of function, determines the sensitivity of breast cancer cells to mTOR inhibitory drugs. Oncogene 30: 3222–3233. 21358673 10.1038/onc.2011.42

[14] Gera JF, Mellinghoff IK, Shi Y, Rettig MB, Tran C, et al (2004) AKT activity determines sensitivity to mammalian target of rapamycin (mTOR) inhibitors by regulating cyclin D1 and c-myc expression. J Biol Chem 279: 2737–2746. 14576155 10.1074/jbc.M309999200

[15] Di Nicolantonio F, Arena S, Tabernero J, Grosso S, Molinari F, et al (2010) Deregulation of the PI3K and KRAS signaling pathways in human cancer cells determines their response to everolimus. J Clin Invest 120: 2858–2866. 20664172 10.1172/JCI37539 PMC2912177

[16] Liao X, Morikawa T, Lochhead P, Imamura Y, Kuchiba A, et al (2012) Prognostic Role of PIK3CA Mutation in Colorectal Cancer: Cohort Study and Literature Review. Clin Cancer Res 18: 2257–2268. 22357840 10.1158/1078-0432.CCR-11-2410 PMC3628835

[17] Velho S, Oliveira C, Ferreira A, Ferreira AC, Suriano G, et al (2005) The prevalence of PIK3CA mutations in gastric and colon cancer. Eur J Cancer 41: 1649–1654. 15994075 10.1016/j.ejca.2005.04.022

[18] American Cancer Society. (2011) Cancer facts & figures. Atlanta, GA: The Society. pp. v.

[19] Serkova N, Jacobsen W, Niemann CU, Litt L, Benet LZ, et al (2001) Sirolimus, but not the structurally related RAD (everolimus), enhances the negative effects of cyclosporine on mitochondrial metabolism in the rat brain. Br J Pharmacol 133: 875–885. 11454661 10.1038/sj.bjp.0704142 PMC1572850

[20] Serkova NJ, Niemann CU (2006) Pattern recognition and biomarker validation using quantitative 1H-NMR-based metabolomics. Expert Rev Mol Diagn 6: 717–731. 17009906 10.1586/14737159.6.5.717

[21] Serkova NJ, Glunde K (2009) Metabolomics of cancer. Methods Mol Biol 520: 273–295. 19381962 10.1007/978-1-60327-811-9_20

[22] Klawitter J, Gottschalk S, Hainz C, Leibfritz D, Christians U, et al (2010) Immunosuppressant neurotoxicity in rat brain models: oxidative stress and cellular metabolism. Chem Res Toxicol 23: 608–619. 20148532 10.1021/tx900351q PMC2841482

[23] Boros LG, Boros TF (2007) Use of metabolic pathway flux information in anticancer drug design. Ernst Schering Found Symp Proc: 189–203. 10.1007/2789_2008_094 18811058

[24] Koch G, Walz A, Lahu G, Schropp J (2009) Modeling of tumor growth and anticancer effects of combination therapy. Journal of Pharmacokinetics and Pharmacodynamics 36: 179–197. 19387803 10.1007/s10928-009-9117-9

[25] O’Donnell A, Faivre S, Burris HA, 3rd, Rea D, Papadimitrakopoulou V, et al (2008) Phase I pharmacokinetic and pharmacodynamic study of the oral mammalian target of rapamycin inhibitor everolimus in patients with advanced solid tumors. J Clin Oncol 26: 1588–1595. 18332470 10.1200/JCO.2007.14.0988

[26] Okamoto I, Doi T, Ohtsu A, Miyazaki M, Tsuya A, et al (2010) Phase I clinical and pharmacokinetic study of RAD001 (everolimus) administered daily to Japanese patients with advanced solid tumors. Jpn J Clin Oncol 40: 17–23. 19783551 10.1093/jjco/hyp120 PMC2800315

[27] (2010) Irinotecan Full Prescribing Information. In: Pfizer I, editor.

[28] Delbaldo C, Pierga JY, Dieras V, Faivre S, Laurence V, et al (2005) Pharmacokinetic profile of cetuximab (Erbitux) alone and in combination with irinotecan in patients with advanced EGFR-positive adenocarcinoma. Eur J Cancer 41: 1739–1745. 16051481 10.1016/j.ejca.2005.04.029

[29] Slatter JG, Schaaf LJ, Sams JP, Feenstra KL, Johnson MG, et al (2000) Pharmacokinetics, metabolism, and excretion of irinotecan (CPT-11) following I.V. infusion of [(14)C]CPT-11 in cancer patients. Drug Metab Dispos 28: 423–433. 10725311

[30] Saunders MP, Wilson R, Peeters M, Smith R, Godwood A, et al (2009) Vandetanib with FOLFIRI in patients with advanced colorectal adenocarcinoma: results from an open-label, multicentre Phase I study. Cancer Chemother Pharmacol 64: 665–672. 19184020 10.1007/s00280-008-0914-4

[31] O’Reilly T, McSheehy PM, Kawai R, Kretz O, McMahon L, et al (2010) Comparative pharmacokinetics of RAD001 (everolimus) in normal and tumor-bearing rodents. Cancer Chemother Pharmacol 65: 625–639. 19784839 10.1007/s00280-009-1068-8

[32] Laplanche R, Meno-Tetang GM, Kawai R (2007) Physiologically based pharmacokinetic (PBPK) modeling of everolimus (RAD001) in rats involving non-linear tissue uptake. J Pharmacokinet Pharmacodyn 34: 373–400. 17431753 10.1007/s10928-007-9051-7

[33] Lane HA, Wood JM, McSheehy PM, Allegrini PR, Boulay A, et al (2009) mTOR inhibitor RAD001 (everolimus) has antiangiogenic/vascular properties distinct from a VEGFR tyrosine kinase inhibitor. Clin Cancer Res 15: 1612–1622. 19223496 10.1158/1078-0432.CCR-08-2057

[34] Mordant P, Loriot Y, Leteur C, Calderaro J, Bourhis J, et al (2010) Dependence on phosphoinositide 3-kinase and RAS-RAF pathways drive the activity of RAF265, a novel RAF/VEGFR2 inhibitor, and RAD001 (Everolimus) in combination. Mol Cancer Ther 9: 358–368. 20124452 10.1158/1535-7163.MCT-09-1014

[35] Guichard S, Chatelut E, Lochon I, Bugat R, Mahjoubi M, et al (1998) Comparison of the pharmacokinetics and efficacy of irinotecan after administration by the intravenous versus intraperitoneal route in mice. Cancer Chemother Pharmacol 42: 165–170. 9654118 10.1007/s002800050801

[36] Kaneda N, Nagata H, Furuta T, Yokokura T (1990) Metabolism and pharmacokinetics of the camptothecin analogue CPT-11 in the mouse. Cancer Res 50: 1715–1720. 2306725

[37] Stewart CF, Zamboni WC, Crom WR, Houghton PJ (1997) Disposition of irinotecan and SN-38 following oral and intravenous irinotecan dosing in mice. Cancer Chemother Pharmacol 40: 259–265. 9219511 10.1007/s002800050656

[38] Zamboni WC, Houghton PJ, Thompson J, Cheshire PJ, Hanna SK, et al (1998) Altered irinotecan and SN-38 disposition after intravenous and oral administration of irinotecan in mice bearing human neuroblastoma xenografts. Clin Cancer Res 4: 455–462. 9516936

[39] Zamboni WC, Stewart CF, Cheshire PJ, Richmond LB, Hanna SK, et al (1998) Studies of the efficacy and pharmacology of irinotecan against human colon tumor xenograft models. Clin Cancer Res 4: 743–753. 9533544

[40] Messersmith WA, Laheru DA, Senzer NN, Donehower RC, Grouleff P, et al (2004) Phase I trial of irinotecan, infusional 5-fluorouracil, and leucovorin (FOLFIRI) with erlotinib (OSI-774): early termination due to increased toxicities. Clin Cancer Res 10: 6522–6527. 15475439 10.1158/1078-0432.CCR-04-0746

[41] Gabrielsson J, Green AR, Van der Graaf PH (2010) Optimising in vivo pharmacology studies–Practical PKPD considerations. J Pharmacol Toxicol Methods 61: 146–156. 20153442 10.1016/j.vascn.2010.02.002

[42] Serkova NJ, Spratlin JL, Eckhardt SG (2007) NMR-based metabolomics: translational application and treatment of cancer. Curr Opin Mol Ther 9: 572–585. 18041668

[43] Manegold PC, Paringer C, Kulka U, Krimmel K, Eichhorn ME, et al (2008) Antiangiogenic therapy with mammalian target of rapamycin inhibitor RAD001 (Everolimus) increases radiosensitivity in solid cancer. Clin Cancer Res 14: 892–900. 18245553 10.1158/1078-0432.CCR-07-0955

[44] Joshi SS, Jackson JD, Sharp JG (1989) Comparison of the growth and metastasis of four human intestinal tumor cell line xenografts. Tumour Biol 10: 117–125. 2762735 10.1159/000217607

[45] Yao K, Gietema JA, Shida S, Selvakumaran M, Fonrose X, et al (2005) In vitro hypoxia-conditioned colon cancer cell lines derived from HCT116 and HT29 exhibit altered apoptosis susceptibility and a more angiogenic profile in vivo. Br J Cancer 93: 1356–1363. 16333244 10.1038/sj.bjc.6602864 PMC2361533

[46] Mohseni M, Park BH (2010) PIK3CA and KRAS mutations predict for response to everolimus therapy: now that’s RAD001. J Clin Invest 120: 2655–2658. 20664174 10.1172/JCI44026 PMC2912204

[47] Richman SD, Seymour MT, Chambers P, Elliott F, Daly CL, et al (2009) KRAS and BRAF mutations in advanced colorectal cancer are associated with poor prognosis but do not preclude benefit from oxaliplatin or irinotecan: results from the MRC FOCUS trial. J Clin Oncol 27: 5931–5937. 19884549 10.1200/JCO.2009.22.4295

[48] Yao JC, Lombard-Bohas C, Baudin E, Kvols LK, Rougier P, et al (2010) Daily oral everolimus activity in patients with metastatic pancreatic neuroendocrine tumors after failure of cytotoxic chemotherapy: a phase II trial. J Clin Oncol 28: 69–76. 19933912 10.1200/JCO.2009.24.2669 PMC4295034

[49] Calvo E, Escudier B, Motzer RJ, Oudard S, Hutson TE, et al (2012) Everolimus in metastatic renal cell carcinoma: Subgroup analysis of patients with 1 or 2 previous vascular endothelial growth factor receptor-tyrosine kinase inhibitor therapies enrolled in the phase III RECORD-1 study. Eur J Cancer 48: 333–339. 22209391 10.1016/j.ejca.2011.11.027

[50] O’Reilly T, McSheehy PM, Wartmann M, Lassota P, Brandt R, et al (2011) Evaluation of the mTOR inhibitor, everolimus, in combination with cytotoxic antitumor agents using human tumor models in vitro and in vivo. Anticancer Drugs 22: 58–78. 20890178 10.1097/CAD.0b013e3283400a20

