# Supplementary material for: Utilization of Quantitative In Vivo Pharmacology Approaches to Assess Combination Effects of Everolimus and Irinotecan in Mouse Xenograft Models of Colorectal Cancer
Source: PLoS One. 2013 Mar 8;8(3):e58089. doi: 10.1371/journal.pone.0058089 (PMC3592886; doi:10.1371/journal.pone.0058089)
Supplement: Supplement S1 — Supporting methods. (PDF) [file pone.0058089.s001.pdf]

Supplement A

SUPPLEMENTAL METHODS:

SA.1. Everolimus LC/MS/MS Assay.

Positive ion electrospray ionization (ESI) mass spectra were obtained with a MDS Sciex 3200 Q-TRAP triple quadrupole mass spectrometer (Applied Biosystems, Inc., Foster City, CA) with a turbo ionspray source interfaced to an Agilent 1200 Series Binary Pump SL HPLC system (Santa Clara, CA). Samples were chromatographed with an Atlantis Hilic, 3.0 µm, 4.6 x 50 mm column (Waters Corporation, Milford, MA) protected by a C18 guard cartridge, 4.0 x 2.0 mm (Phenomenex, Torrance, CA). An isocratic mobile phase consisting of 85% acetonitrile and 15% ammonium acetate/0.1% acetic acid was utilized with a flow rate was 1.0 mL/min, sample injection volume of 60 µL, column temperature of 50°C and an analysis run time of 3.5 min.

The mass spectrometer settings were optimized as follows: turbo ionspray temperature, 600 °C; ion spray voltage, 5500 V; declustering potential (DP), 30 V; entrance potential (EP), 8 V; collision energy (CE), 24 V; collision cell entrance potential (CEP), 30 V; collision cell exit potential (CXP), 10 V; curtain gas, N_2, (CUR), 30 units; collision gas, N_2, (CAD), low; nebulizer gas, N_2, 10 units; and auxiliary gas, N_2, 20 units. Samples were quantified in the MRM mode monitoring ion transitions *m/z* 975.6 → 908.6 amu for RAD-001. Each ion transition was integrated for 200 ms and Q_1 and Q_3 were both operated in unit resolution mode.

SA.2. NMR-Based Metabolomics.

Briefly, following centrifugation, supernatants (containing hydrophilic metabolites) and tissue pellets (lipids) were separated, neutralized to pH=7 and lyophilized overnight. Tissue hydrophilic extracts were re-dissolved in 500 µL deuterium oxide (D_2O, Cambridge Isotopes, Cambridge, MA) and lipids in 1 mL deuterated methanol/ chloroform mixture (2:1 vol/vol).

All one-dimensional nuclear magnetic resonance (NMR) spectra were obtained using Bruker 300 (^{31}P-NMR) and 500 MHz (water-suppressed ^{1}H- and ^{13}C-NMR) Avance

spectrometers with 5-mm probe heads (Bruker Medical, Fremont, CA). Trimethylsilyl propionic-2,2.3.3-d_4 acid (TSP, chemical shift 0 ppm) and methylene diphosphoric acid (MDP, 18 ppm) in sealed glass capillaries were used as external standards for the quantification of ^{1}H- and ^{31}P-metabolites according to:

$$C_X = \frac{I_X : N_X : C}{I : 9} xV : M$$

where C_X is metabolite concentration, I_X is the integral of the ^{1}H- or 31P-metabolite, N_X is the number of protons/ phosphorus in metabolite peak, C is TSP or MDA concentration, I is external standard integral, V is the volume of the extract and M is the weight of the tumor tissue. For calculation of ^{13}C-labeled metabolites (from [1-^{13}C]glucose metabolism), the ^{1}H-"satellite" peak of [3-^{13}C]lactate (1.22 ppm/ 21 ppm on ^{1}H/ ^{13}C-NMR spectra) was used as an internal standard for ^{13}C-NMR spectra(22). All spectra were acquired using Bruker TopSpin and processed using Bruker 1DWINNMR softwares. All metabolite concentrations are given as µmol per gram tissue.

SA.3. Synergy Modeling.

The combination of everolimus and irinotecan therapy was determined by mathematical modeling of the tumor growth profiles of individual tumors similarly to a previously published method (24). All HT29 and HCT116 control (vehicle treated) tumors were individually fit to the following growth equation:

$$T'(t) = \frac{2\lambda_0 \lambda_1 T(t)}{\lambda_1 + 2\lambda_0 T(t)} \ , \quad T(0) = T_0$$

where T is the tumor volume (cm^3) with respect to time, λ_0 is the exponential tumor growth parameter, λ_1 is the linear growth parameter and T_0 is the estimated initial tumor volume (at time = 0). The individual parameters (λ_0, λ_1 and T_0) were averaged and the final parameter values

are presented in Tables 1 and 2 in Supplement B. Individual fits of the model to the data can also be found in Supplement B. The average growth terms for each tumor type were then fixed at the average parameter value and used to determine the single agent effects (k_x) of each compound by fitting the following equation:

$$T'(t) = \frac{2\lambda_0\lambda_1 T(t)}{\lambda_1 + 2\lambda_0 T(t)} - k_x c_x(t)T(t), \quad T(0) = T_0$$

where $c_x(t)$ is the concentration of drug x with respect to time. Individual tumors were modeled and the average value of k_x determined (Supplement B Tables 1 & 2) for each single agent treatment. This value was used to fit the following equation to the combination tumor growth data to determine the degree of interaction between the two drugs.

$$T'(t) = \frac{2\lambda_0\lambda_1 T(t)}{\lambda_1 + 2\lambda_0 T(t)} - [\, k_1 c_1(t)T(t) + \, k_2 c_2(t)T(t)\,\psi], \quad T(0) = T_0$$

ψ, the interaction term, was determined for each individual tumor for each tumor type and the data is presented in Table 2 in the main text and individual fits can be found in Supplement B. We determined that ψ values greater than 1.3 are synergistic, between 1.3 and 0.7 are additive, between 0.7 and 0 are less than additive, and negative values for ψ are antagonistic. These ranges were selected based on the model fits. The CV% for the model fits ranged from 15 to 50 with an average CV% of approximately 30%. Therefore we assume an average variance of 30% making the additive range from 1.3 to 0.7 (1 + 30% and 1- 30%).

The pharmacokinetic models used for everolimus and irinotecan and SN38 in the above equations can be found in Supplement B. Data used to fit the everolimus pharmacokinetics was obtained in the study presented here. In the above equations, for irinotecan treatments, SN38 concentration was used to determine the effect since it is the active agent. The data used to fit the irinotecan and SN38 pharmacokinetics was obtained from previous publications (37-39).
