# Supplementary material for: Utilization of Quantitative In Vivo Pharmacology Approaches to Assess Combination Effects of Everolimus and Irinotecan in Mouse Xenograft Models of Colorectal Cancer
Source: PLoS One. 2013 Mar 8;8(3):e58089. doi: 10.1371/journal.pone.0058089 (PMC3592886; doi:10.1371/journal.pone.0058089)

Supplementary Data B

Pharmacokinetic and Synergy Modeling

In vivo Synergy Model

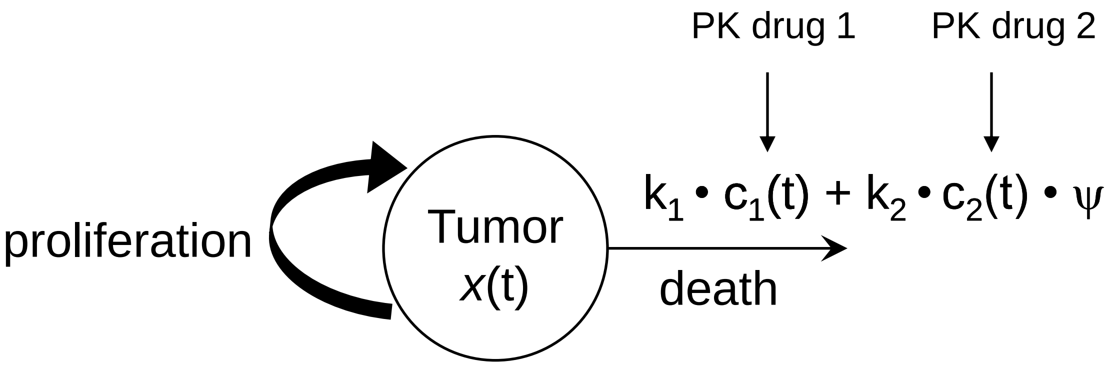

$$growth = \frac{2\lambda_0 \lambda_1 x(t)}{\lambda_1 + 2\lambda_0 x(t)}, \quad x(0) = T_0$$

1 Drug $$death = k_1 c_{drug1}(t) x(t)$$

2 Drugs $$death = [k_1 c_{drug1}(t) + k_2 c_{drug2}(t) \bullet \psi] x(t)$$

Everolimus PK

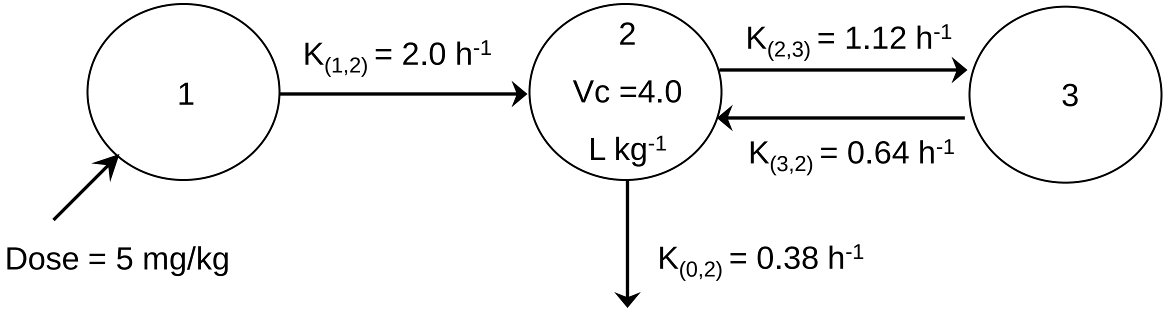

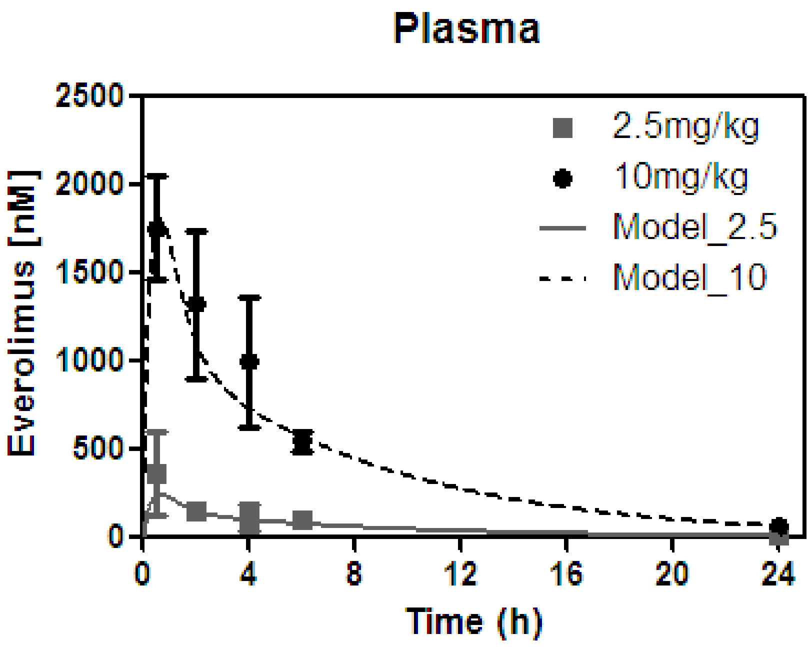

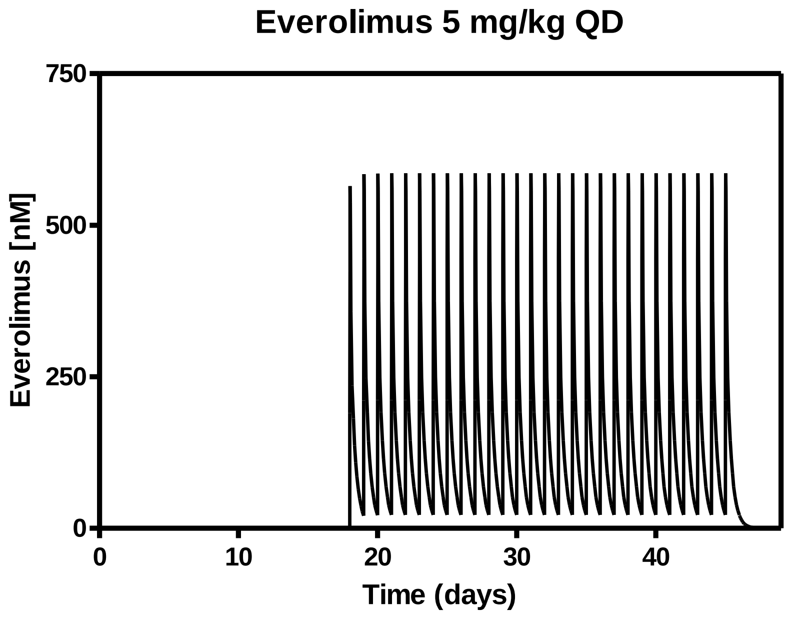

Irinotecan/SN-38

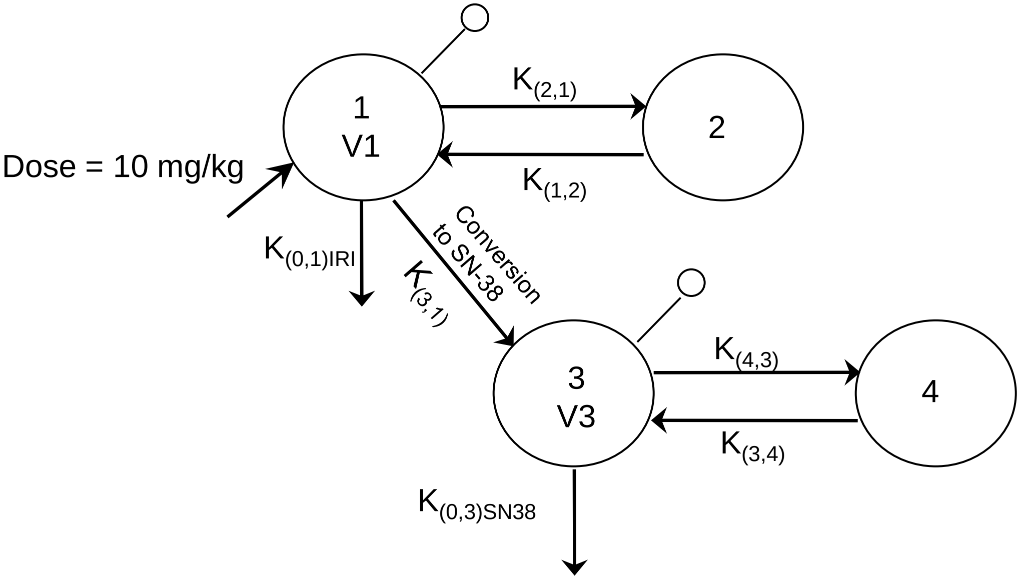

Parameter	Value
V1 (L/kg)	2.8
V3 (L/kg)	2.7
k(0,1) (hr-1)	0.25
k(0,3) (hr-1)	4.67
k(1,2) (hr-1)	1.15
k(2,1) (hr-1)	0.87
k(3,1) (hr-1)	1.91
k(3,4) (hr-1)	1.38
k(4,3) (hr-1)	5.22

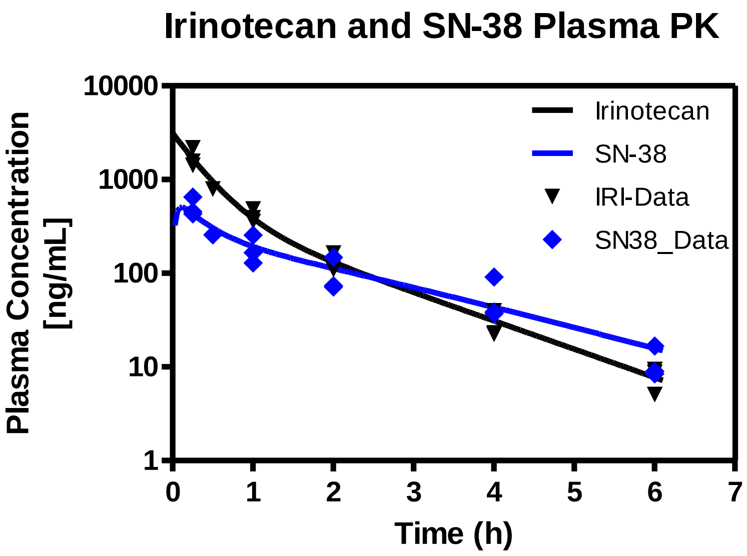

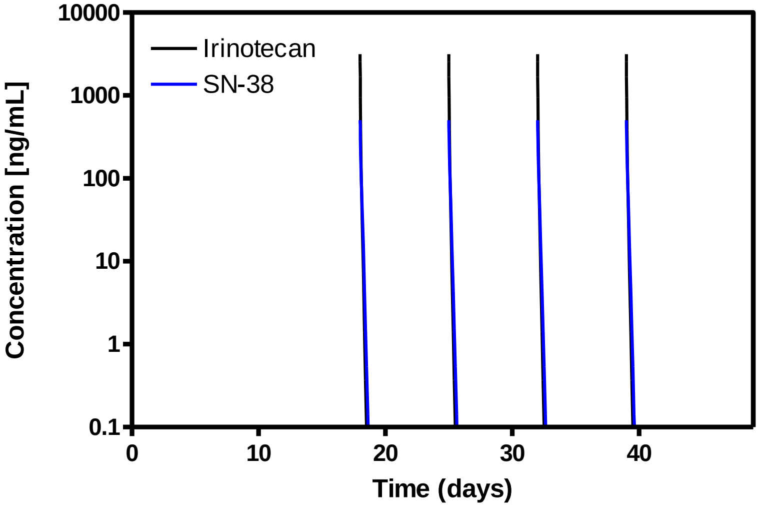

Table SB.1. HT29 (*BRAF* and *PIK3CA* mutant) Parameter Values

Parameter	Control	IRI	RAD
λ_0 [day^{-1}] (SD)	0.067 (0.045)		
λ_1 [cm^3/day] (SD)	0.203 (0.148)		
T_0 [cm^3] (SD)	0.042 (0.027)	0.037 (0.012)	0.042 (0.018)
k_1 [mL/ng*day^{-1}] (SD)		0.0092 (0.0023)	
k_2 [mL/ng*day^{-1}] (SD)			0.000263 (0.00015)

Table SB.2. HCT116 (*KRAS* and *PIK3CA* mutant) Parameter Values

Parameter	Control	IRI	RAD
λ_0 [day^{-1}] (SD)	0.086 (0.053)		
λ_1 [cm^3/day] (SD)	0.187 (0.185)		
T_0 [cm^3] (SD)	0.019 (0.019)	0.023 (0.007)	0.013 (0.006)
k_1 [mL/ng*day^{-1}] (SD)		0.0149 (0.0086)	
k_2 [mL/ng*day^{-1}] (SD)			0.000482 (0.00011)

HT-29 Control

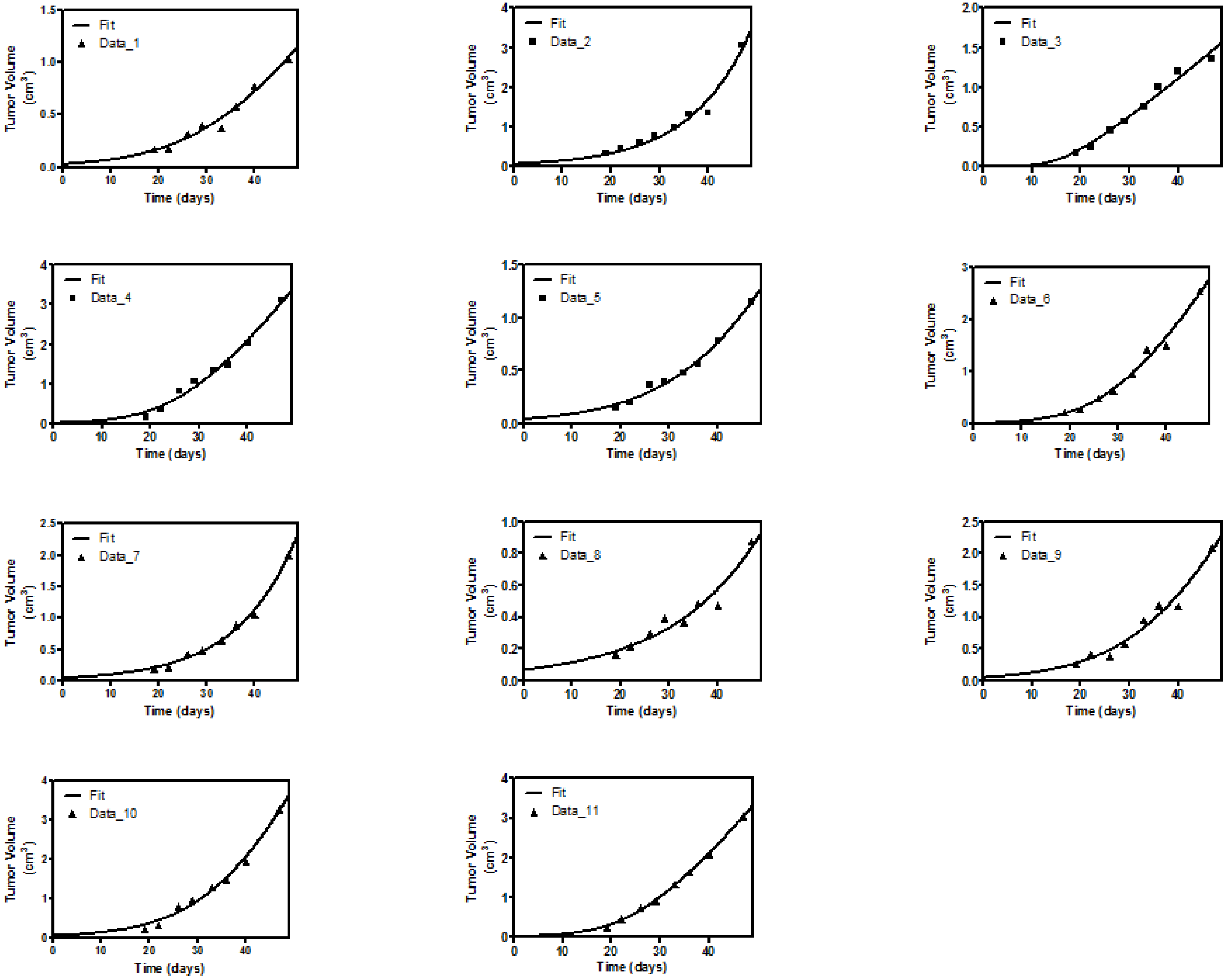

HT-29 Irinotecan

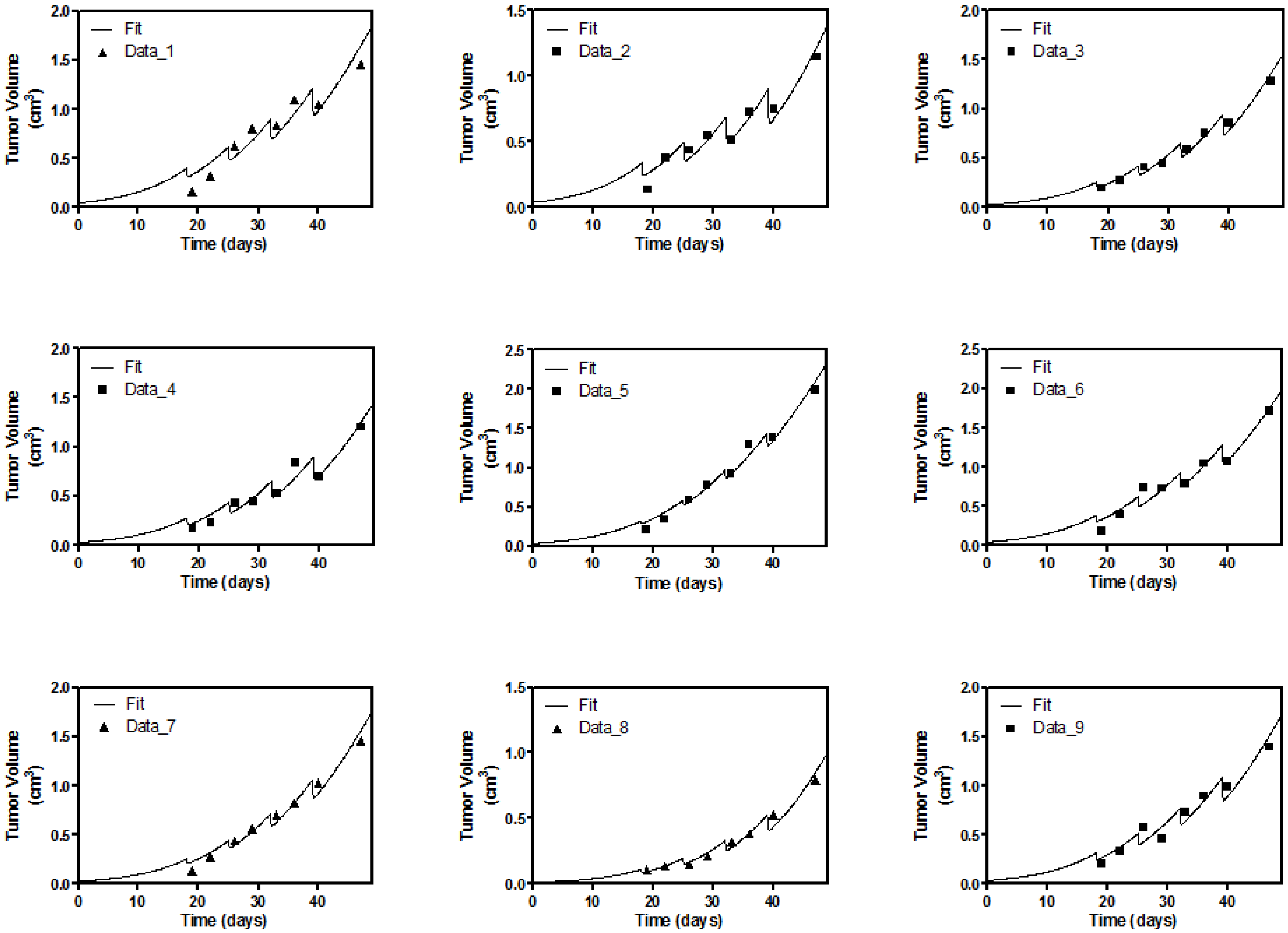

HT-29 RAD001

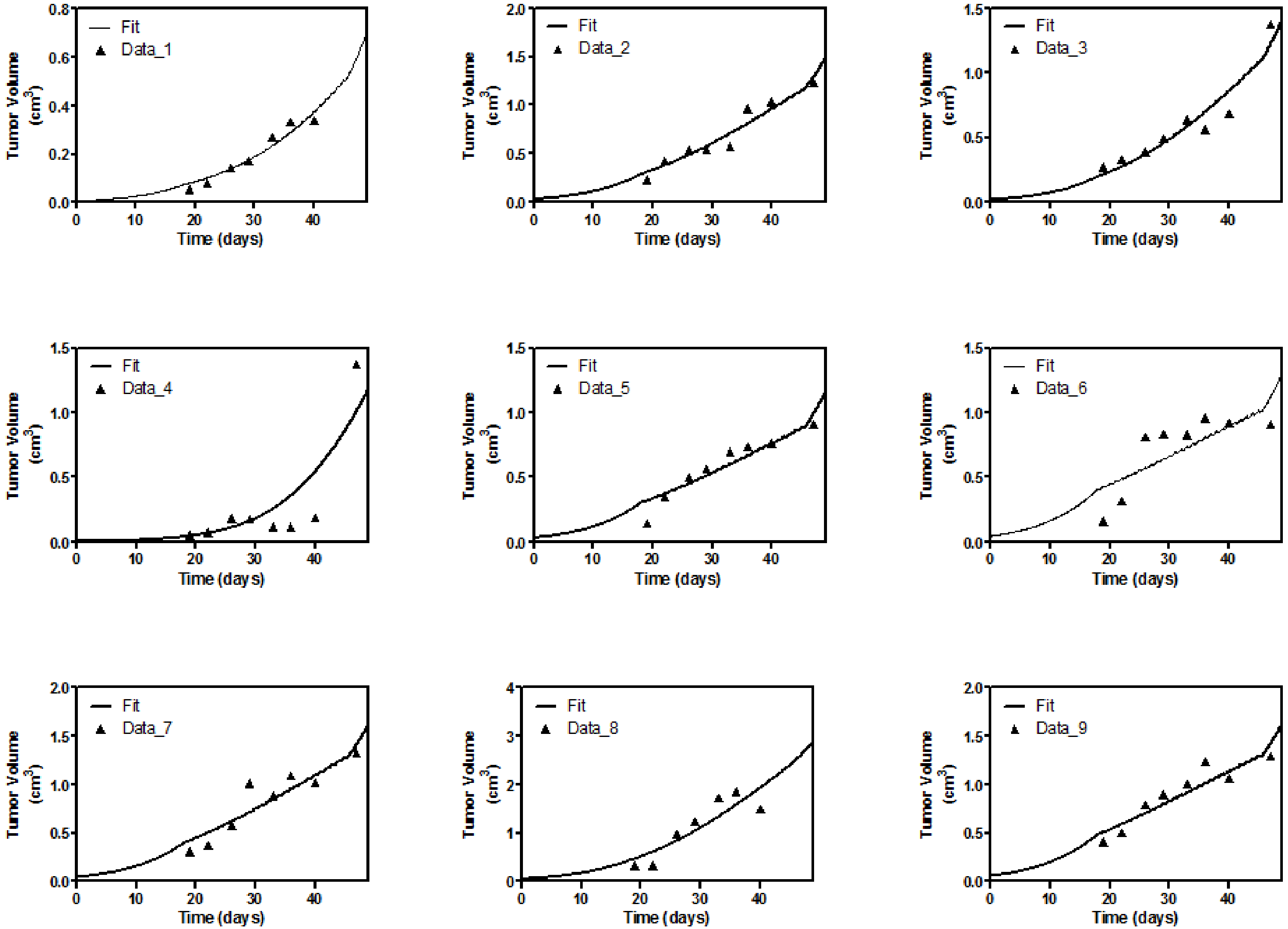

Fit
Data_1
Tumor Volume (cm3)
Time (days)
Fit
Data_2
Tumor Volume (cm3)
Time (days)
Fit
Data_3
Tumor Volume (cm3)
Time (days)
Fit
Data_4
Tumor Volume (cm3)
Time (days)
Fit
Data_5
Tumor Volume (cm3)
Time (days)
Fit
Data_6
Tumor Volume (cm3)
Time (days)
Fit
Data_7
Tumor Volume (cm3)
Time (days)
Fit
Data_8
Tumor Volume (cm3)
Time (days)
Fit
Data_9
Tumor Volume (cm3)
Time (days)

HT-29 RAD + Irinotecan

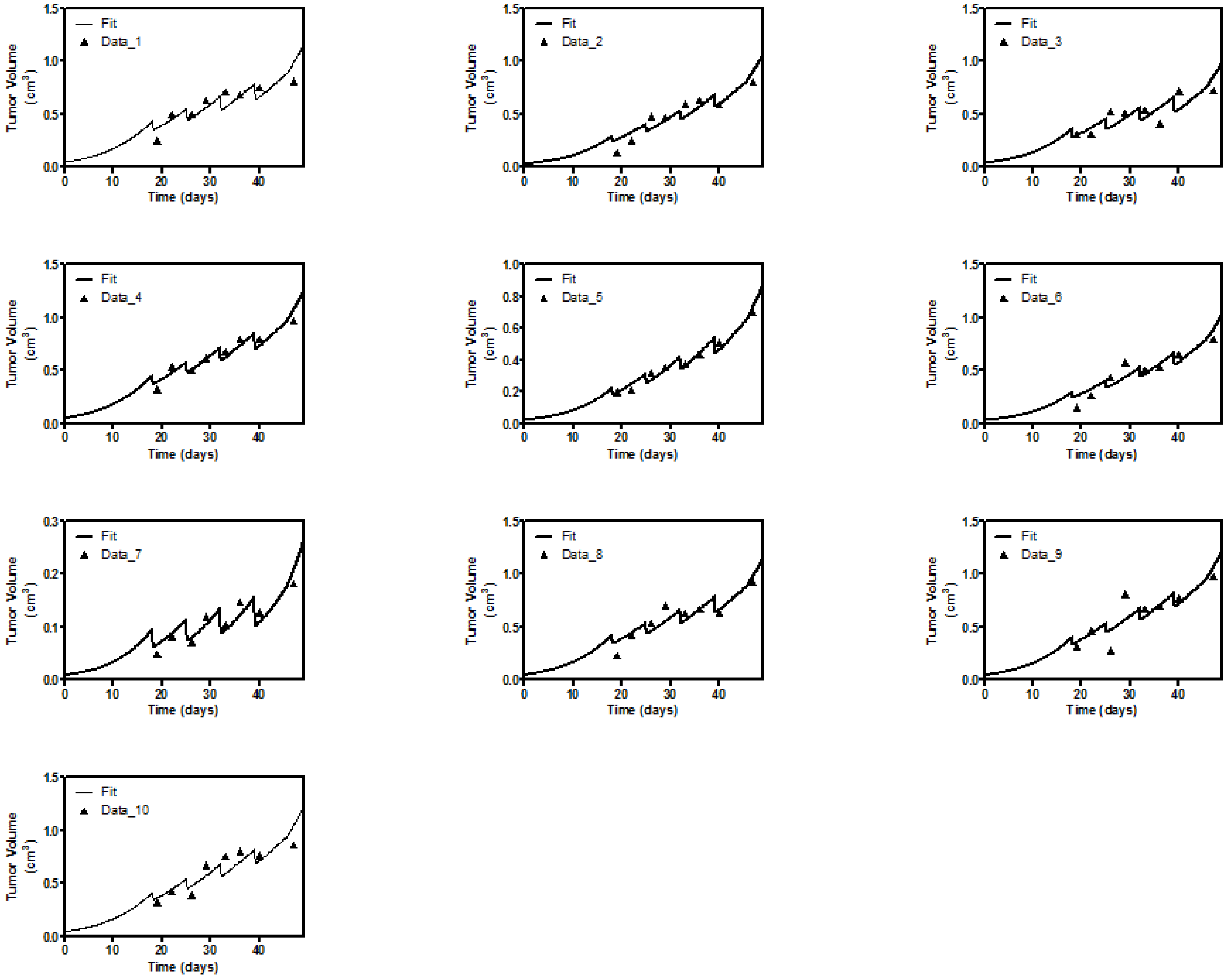

HCT116 Control

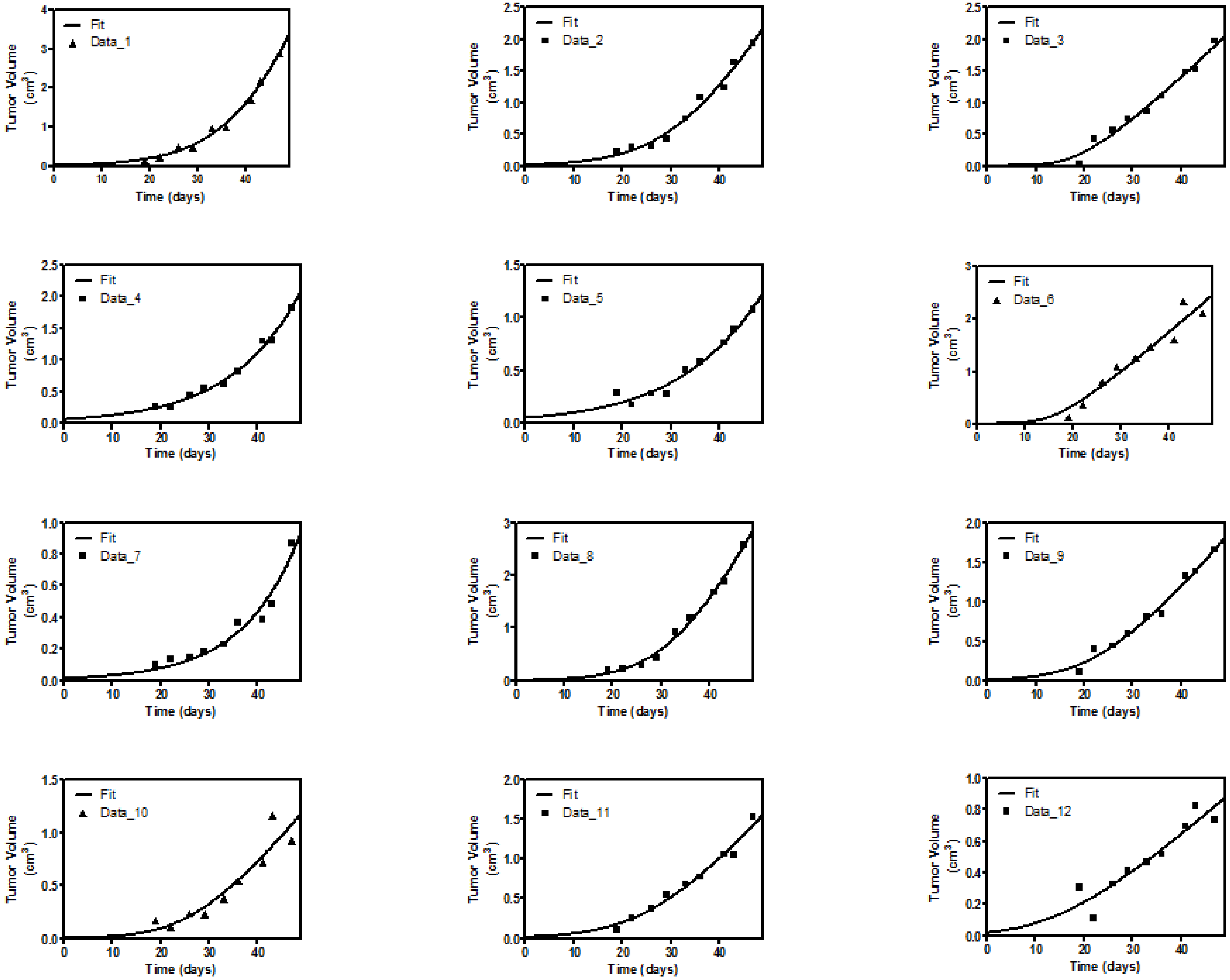

HCT116 Irinotecan

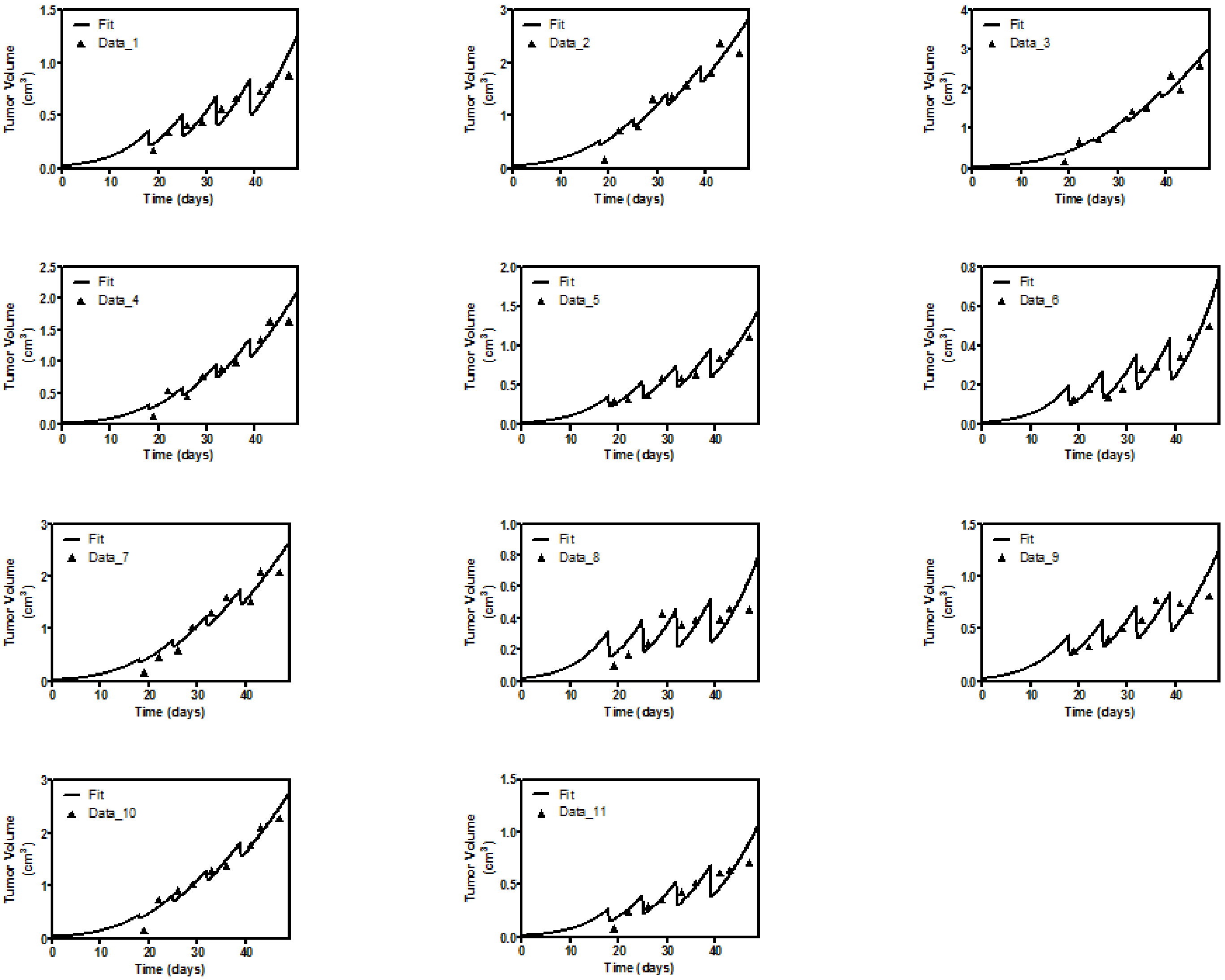

HCT116 RAD001

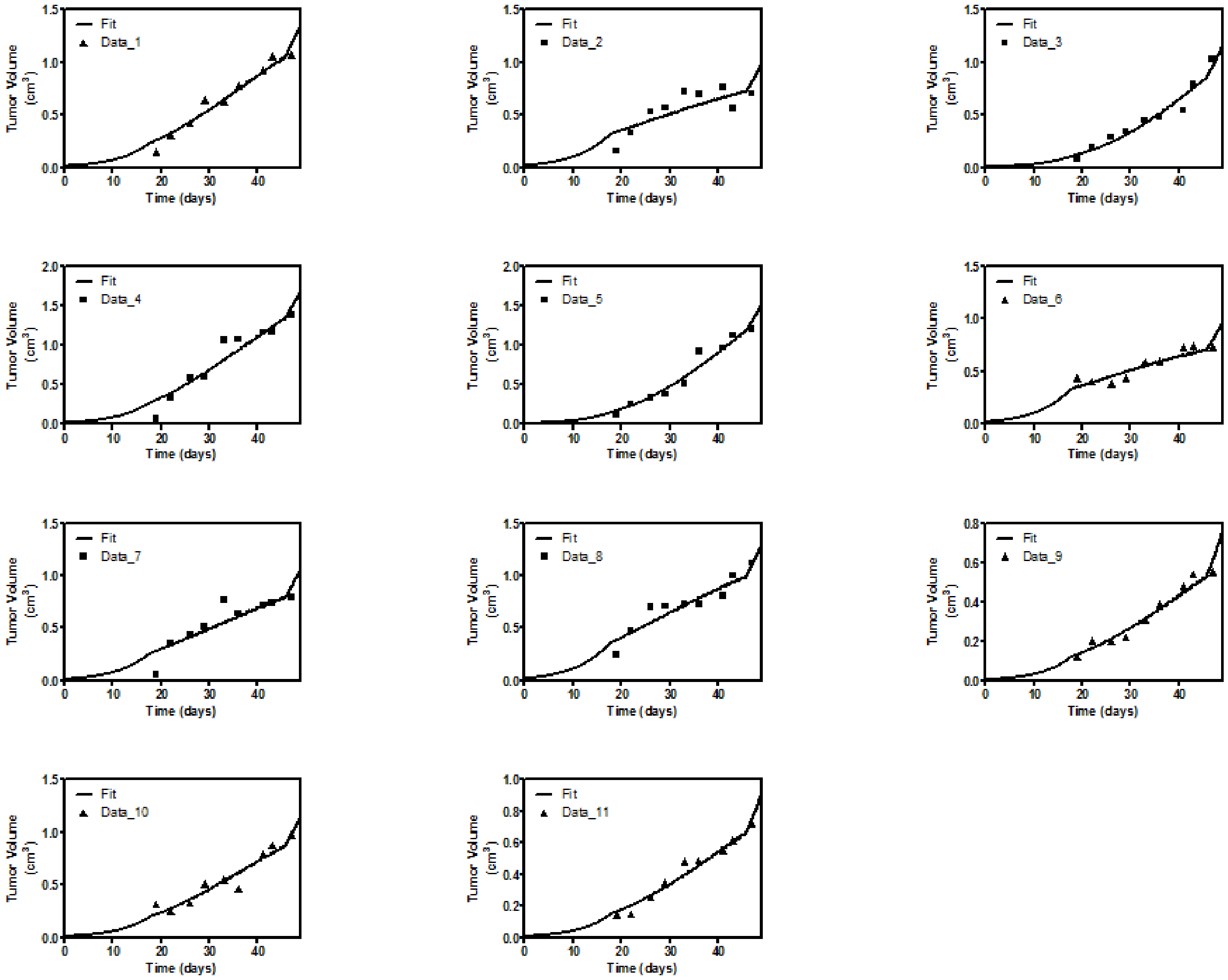

HCT116 RAD + Irinotecan

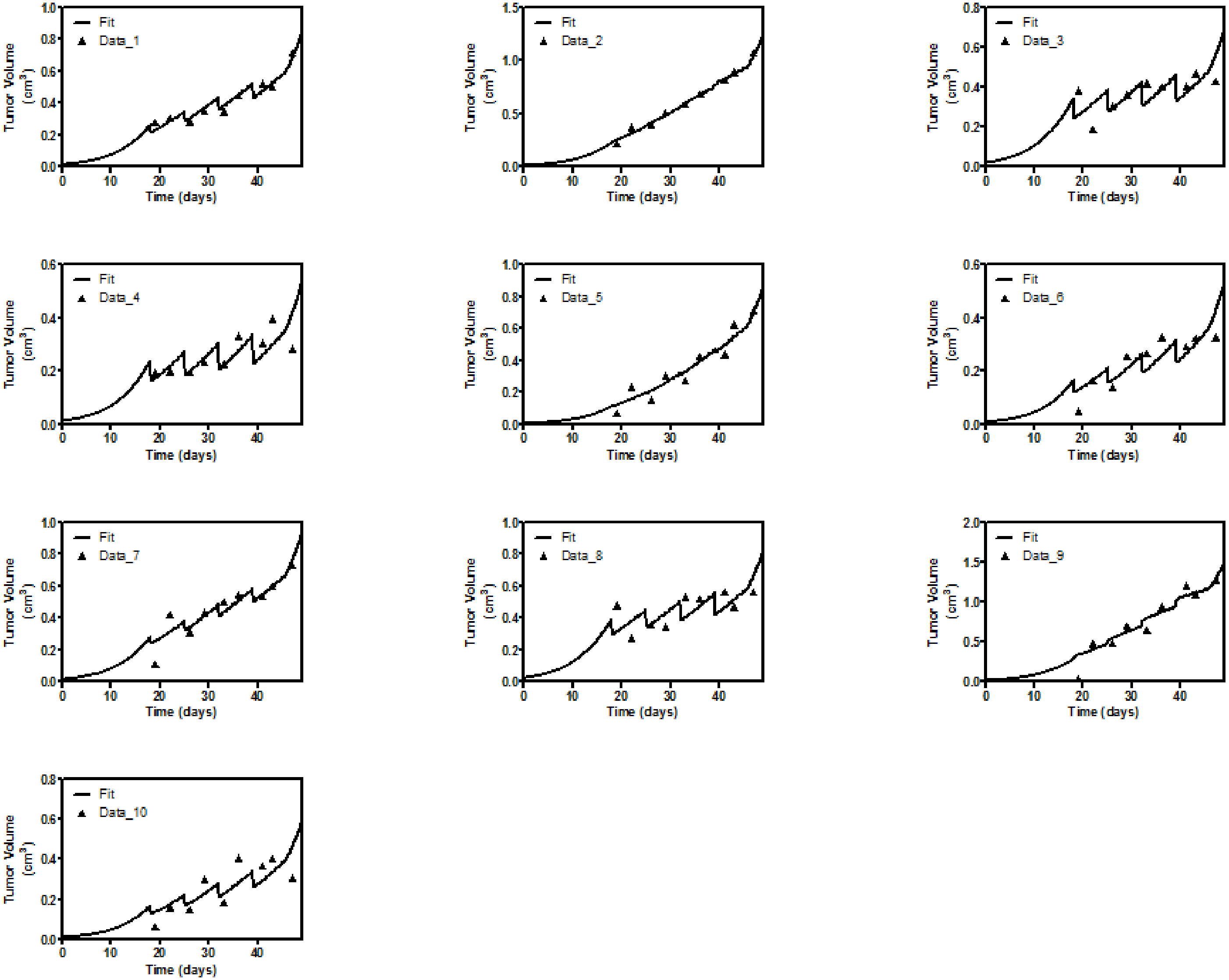

Supplement: Supplement S2 — Pharmacokinetic and Synergy Modeling. (PDF) [file pone.0058089.s002.pdf]
