# Supplementary material for: Utilization of Quantitative In Vivo Pharmacology Approaches to Assess Combination Effects of Everolimus and Irinotecan in Mouse Xenograft Models of Colorectal Cancer
Source: PLoS One. 2013 Mar 8;8(3):e58089. doi: 10.1371/journal.pone.0058089 (PMC3592886; doi:10.1371/journal.pone.0058089)
Supplement: Supplement S3 — Supporting figures & tables. (PDF) [file pone.0058089.s003.pdf]

Supplement C

Supplemental Figures and Tables

Figure SC.1.

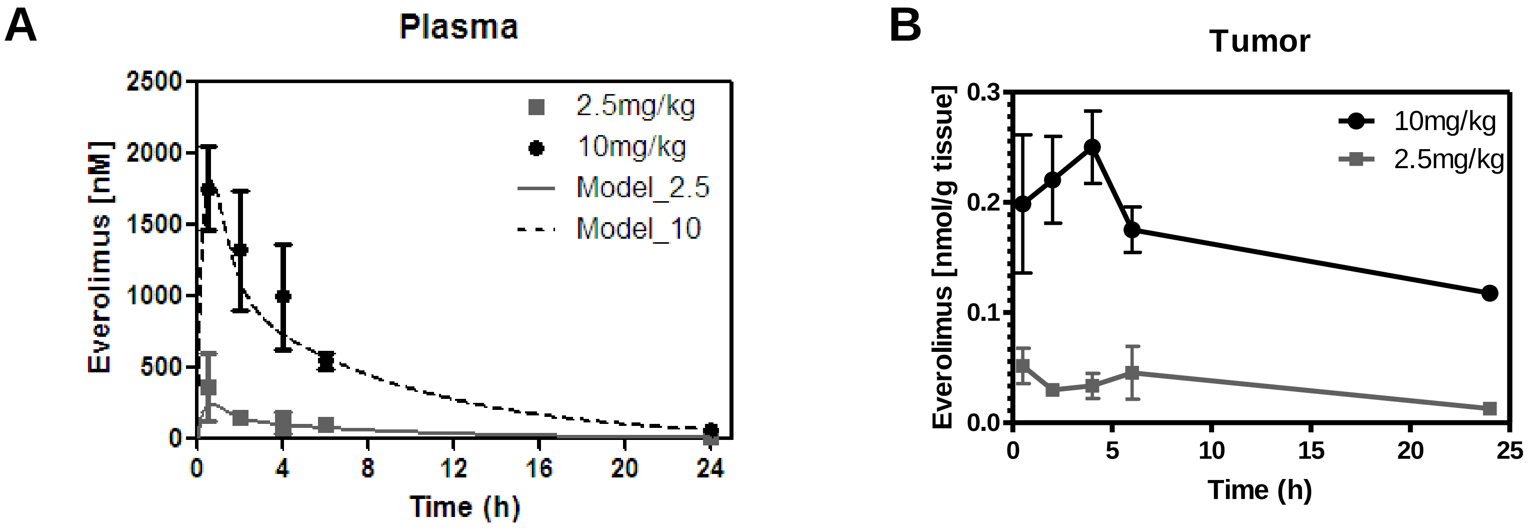

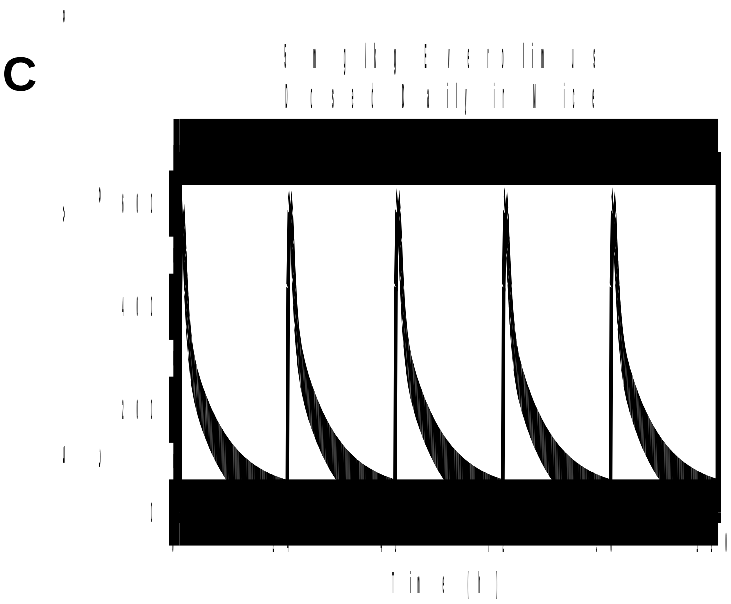

Everolimus plasma (A) and tumor (B) pharmacokinetics were measured in nude mice bearing HT29 xenografts. Mice were dosed with 2.5 (■) or 10 (●) mg/kg everolimus daily for 7 days prior to sample collection. Data represents the average of 3 samples and the lines represent the data fits to a two-compartment model with first-order absorption. The parameter estimates from the fits to the 2.5 and 10 mg/kg data were then used to simulate the steady-state dosing of 5 mg/kg RAD001 (C). Simulations were generated based on daily dosing. Additional details of the study can be found in the main text.

To simulate data at different concentrations between 2.5 and 10 mg/kg the following assumptions were made; (1) everolimus absorption and its' elimination from the central compartment is constant across dose levels, and (2) the volume of the central compartment varies linearly between doses. We believe these assumptions are valid since the pharmacokinetics appeared to be linear between 2.5 and 10 mg/kg doses, and the model fits the measured data at 2.5 and 10 mg/kg doses well and agrees with pharmacokinetic parameters estimated by non-compartmental analysis (Table S1).

Table SC.1. RAD001 plasma and tumor pharmacokinetics in mice determined by non-compartmental analysis

	$t_{½}$ (hr)	T_{max} (hr)	C_{max} (nM)	$AUC_{0\rightarrow24}$ (nM·hr)	V_z/F (L/kg)	CL_{ss}/F (L/hr/kg)
Plasma Pharmacokinetic Parameters in Mice						
2.5 mg/kg	6.0	0.5	365	2005	10.8	1.2
10 mg/kg	5.0	0.5	1758	12098	6.0	0.83
Tumor Pharmacokinetic Parameters						
2.5 mg/kg		0.5	52	742		
10 mg/kg		4.0	250	3925		

Figure SC.2.

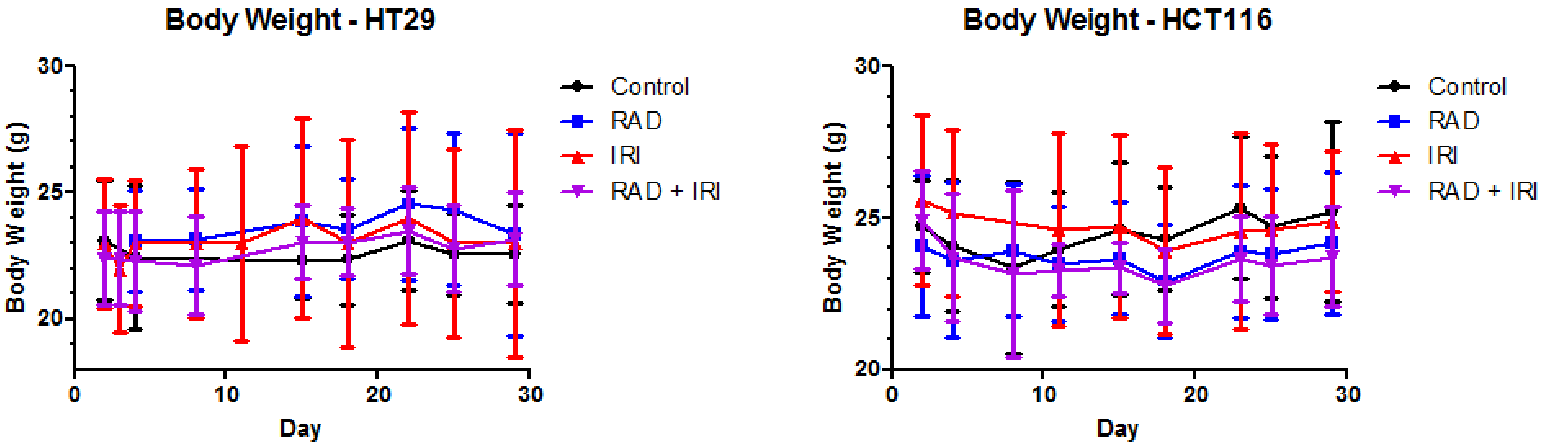

Effect of treatment on bodyweight. In general all compounds were well tolerated and no severe toxicity was observed as measured by bodyweight (< 10% BW loss). Data represent the average ± SD.

Figure SC.3.

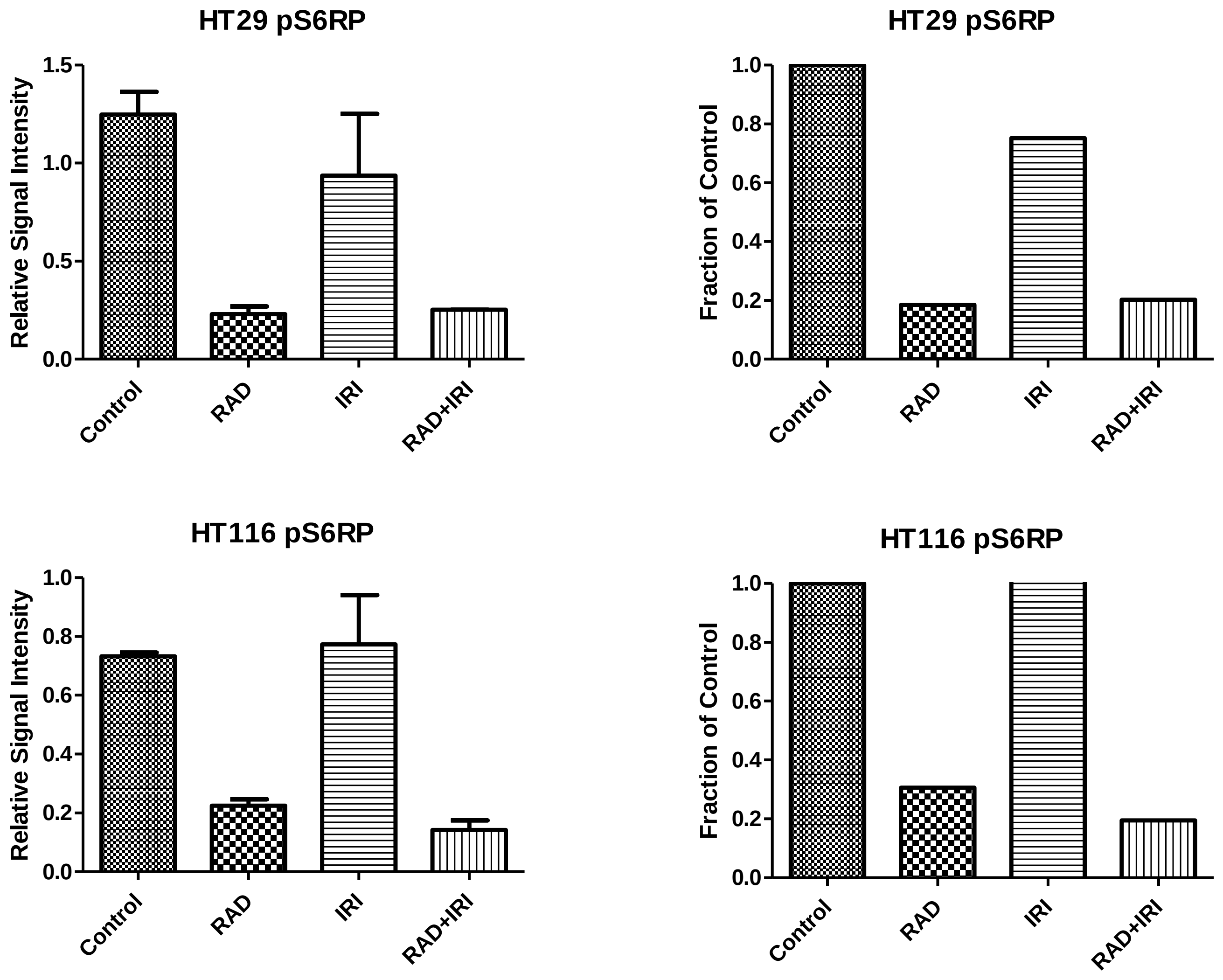

Western blot analysis of pS6RP, total S6RP and actin (loading control) were conducted on HT29 and HCT116 tumor samples taken from animals 24 hours post treatment on the 7th day of the study. Blots were digitized and the ratios of pS6Rp:actin and total S6RP:actin were calculated. Then the ratio of pS6Rp:actin to S6RP:actin were taken and are presented in the graphs on the left. Data presented show the mean $\pm$ SD of 3-4 separate tumors. The graphs on the right represent the average relative signal intensity of pS6RP as a fraction of the control tumors (from vehicle treated animals).

Figure SC.4.

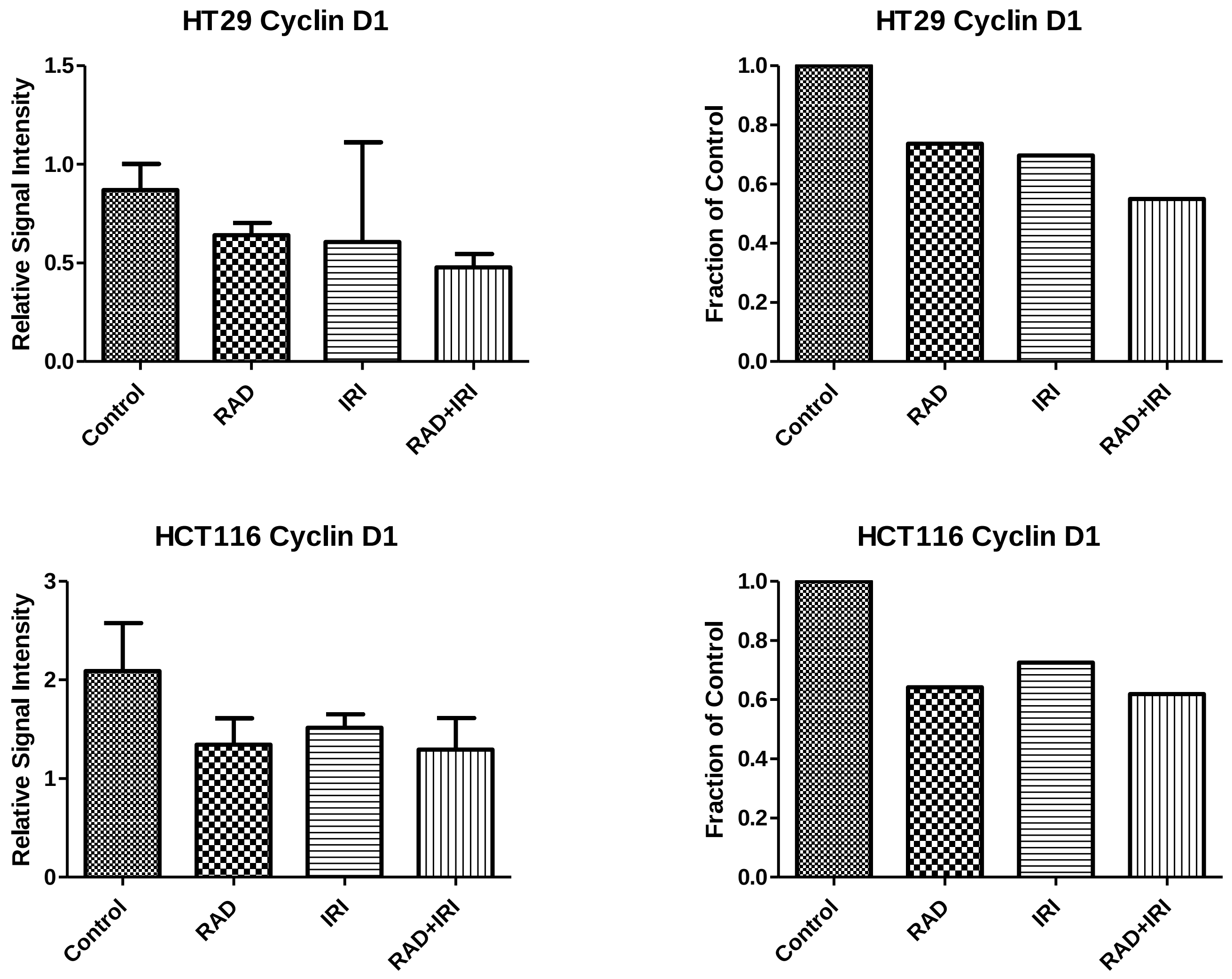

Western blot analysis of cyclin D1 and actin (loading control) were conducted on HT29 and HCT116 tumor samples taken from animals 24 hours post treatment on the 7th day of the study. Blots were digitized and the ratio of cyclin D1:actin were calculated and are presented in the graphs on the left. Data presented show the mean ± SD of 3-4 separate tumors. The graphs on the right represent the average relative signal intensity of cyclin D1 as a fraction of the control tumors (from vehicle treated animals).

Figure SC.5.

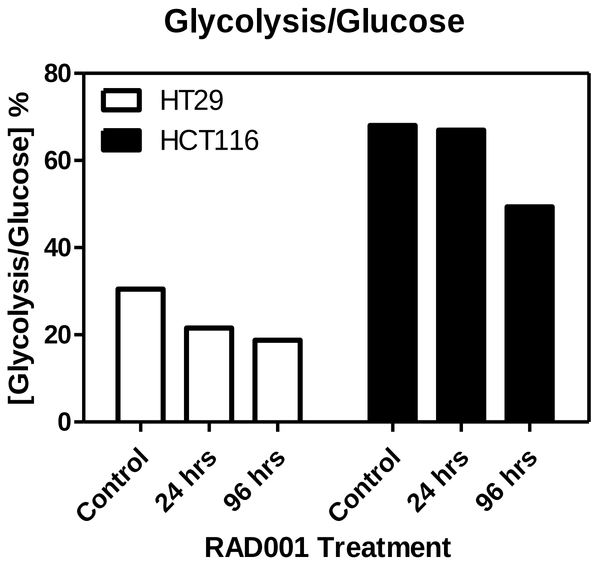

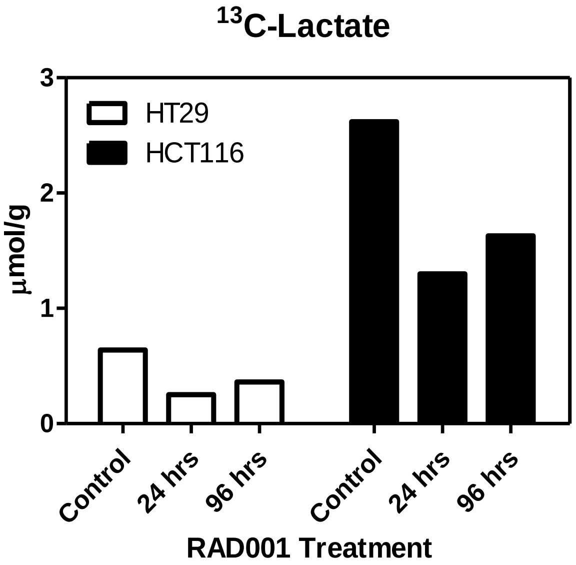

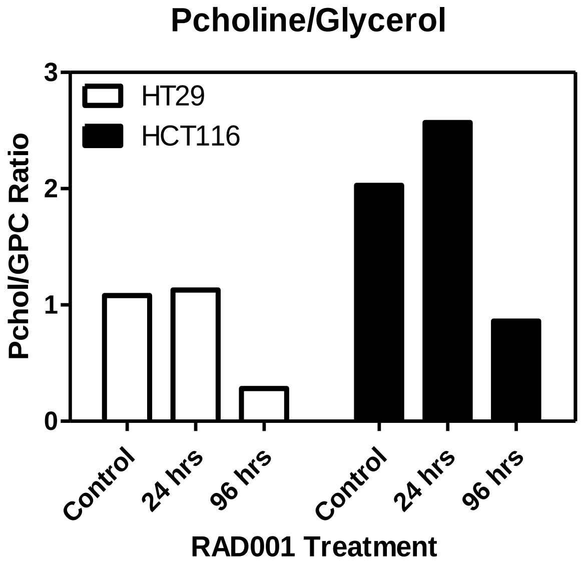

Metabolomics was performed in vitro on HCT116 (*KRAS* mt; *PIK3CA* mt) and HT29 (*BRAF* mt; *PIK3CA* mt) cells after 0 (control), 24 and 96 hours exposure to 30nM RAD001. In both cell lines after 24 and 96 hours glucose uptake was reduced showing a decrease in proliferation.
